# Tailoring aggregation-induced emission in luminescent solar concentrators through controlled polymerization

**DOI:** 10.1038/s42004-025-01700-1

**Published:** 2025-10-17

**Authors:** Elisavet Tatsi, Gaia Roberta Ragno, Andrea Nitti, Chiara Botta, Stefano Turri, Dario Pasini, Gianmarco Griffini

**Affiliations:** 1 Department of Chemistry, Materials and Chemical Engineering “Giulio Natta”, Politecnico di Milano, Milano, Italy; 2 Department of Chemistry, University of Pavia, Pavia, Italy; 3 Institute of Sciences and Chemical Technologies “Giulio Natta” (SCITEC) of CNR, Milano, Italy

**Keywords:** Polymerization mechanisms, Materials for optics

## Abstract

Luminescent solar concentrators (LSCs) hold great potential as versatile energy conversion systems for diverse applications. Here, we explore the covalent incorporation of aggregation-induced emission (AIE) luminogens into polymeric backbones for LSCs. Copolymers based on methyl-methacrylate and an AIE-active monomer (tetraphenyl ethylene methacrylate - TPEMA) are synthesized using free radical (FR) and reversible addition-fragmentation chain transfer (RAFT) polymerizations. By integrating TPEMA into the polymer structure, we exploit its unique emission properties when the AIE*gens* are in close proximity. RAFT polymerization affords copolymers with narrower molecular weight distribution, improved thermal stability, higher glass transition temperature, and optical properties that scale positively with TPEMA content, outperforming FR analogues. RAFT-based LSC devices exhibit more consistent performance, underscoring the importance of controlled polymerization on AIE*gen* response and device behavior. This study demonstrates an effective strategy to enhance LSC response through synthetic control of the macromolecular network and strategic incorporation of AIE*gens* into luminescent polymer matrices.

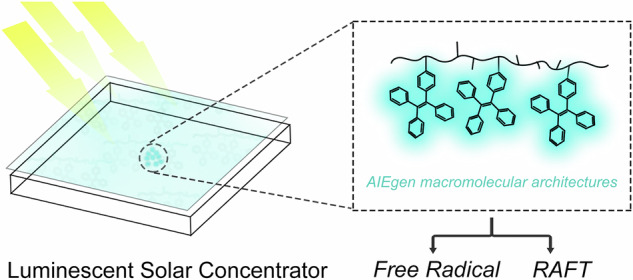

## Abstract (web summary)

Aggregation-induced emission (AIE) luminogens can enhance the photonic response of luminescent solar concentrators (LSCs), but their controlled polymerization to produce macromolecular networks for LSC structures remains underexplored. Here, the authors develop a series of copolymers based on methyl methacrylate and an AIE-active monomer (tetraphenyl ethylene methacrylate) through free radical and reversible addition-fragmentation chain transfer (RAFT) polymerization, showing that RAFT-based LSCs exhibit more consistent device performance.

## Introduction

Luminescent solar concentrators (LSCs) represent a promising energy technology with great potential for integration into a variety of stand-alone and mobile contexts^1–3^. In their most basic configuration, LSCs include a high-transmittance waveguide - generally made of glass or polymeric materials - embedded with (or coated by) one or more luminescent species (luminophores) that absorb incident light and re-emit it at longer wavelengths through photoluminescence (PL). The emitted photons are then confined within the waveguide *via* total internal reflection (TIR) and transported towards the edges of the device, where they can be absorbed by small-area photovoltaic (PV) cells for the light-to-electricity conversion process^4–6^. When compared to PV technologies like perovskite solar cells or dye-sensitized solar cells, LSCs have a more niche focus but hold significant growth potential, especially due to their esthetic versatility, simple device architecture and straightforward manufacturing, which make them particularly interesting for building-integrated photovoltaics (BIPV) and transparent PVs^2,7^. Research on LSCs has bloomed over the years, with a major focus on the development of highly emissive luminophores and highly engineered devices to maximize photonic and PV response^8–13^. However, despite such increased attention, their commercial deployment in large-scale applications still lags behind more mature PV technologies.

One of the most relevant challenges currently faced by LSCs is represented by the optical losses associated with the luminescent species. Among these, reabsorption losses^14–16^ and aggregation-caused quenching (ACQ) are very often encountered in LSC devices incorporating high luminophore concentrations, as typically required for practical applications. Furthermore, chemical-physical compatibility issues between the luminophore and host matrix can also affect device performance, as they may result in restricted ranges of concentration accessible for the emitter. These limitations not only impair the uniform distribution of the luminophore within the host matrix, but also result in non-ideal optical properties, including reduced light absorption, lower TIR efficiency and increased scattering effects, all of which negatively impact the overall waveguiding process^17,18^.

Recent advancements in luminophore design have sought to overcome these limitations by leveraging the aggregation-induced emission (AIE) phenomenon via the incorporation of AIE luminogens (AIE*gens*) in LSC devices^19,20^. Unlike traditional luminescent molecules, which typically suffer from ACQ in the aggregate state, AIE*gens* exhibit enhanced PL when aggregated or in the solid state. This unique property arises from the restriction of intramolecular rotation (RIR), which suppresses non-radiative decay pathways in the aggregate state and leads to a significant increase in luminescence efficiency in molecules typically non-emissive when in solution^18,19,21,22^. The AIE concept was first reported by Ben Zhong Tang and colleagues in 2001^23^. Typical AIE*gens* such as hexaphenylsilole (HPS) and tetraphenylethene (TPE) exhibit a propeller-like structure composed of central silole or olefin stators surrounded by flexible aromatic rotors that minimize π-stacking in the solid state and allow for intense emission.

Over the past few years, several studies have focused on the application of AIE*gens* in LSC devices by dispersion into polymer matrices, aiming to achieve enhanced photoluminescence quantum yield (PLQY) and suppression of ACQ losses^24,25^. While this approach represents a convenient platform to illustrate the ability of AIE luminophores to effectively harvest sunlight at high-optical densities^26^, such host-guest incorporation unavoidably leads to photon scattering within the waveguide when high AIE*gen* concentrations are used. In addition, it does not allow for a rational distribution of the luminophore within the host matrix, potentially leading to luminophore clustering, spatial inhomogeneity and limited control over device response.

An alternative strategy to partially overcome some of these issues is the incorporation of reactive AIE molecules into polymeric networks, so as to build luminescent AIE macromolecular structures characterized by reduced energy losses via the mitigation of scattering and reabsorption^27^. These covalent interactions may also enhance the thermal and photochemical stability of the AIE species, preventing phase separation from the matrix, reducing photobleaching and degradation, and favoring extended LSC lifespan^28,29^. Importantly, AIE polymers can be designed to achieve fine control over the spatial arrangement of the AIE moiety within the macromolecular network by resorting to controlled radical polymerization^30^. Indeed, this synthetic strategy enables well-defined polymeric structures characterized by tunable chain lengths, narrow dispersity and controlled architecture^31^. When applied to luminescent systems, the versatility of this concept has been proven through the preparation of controlled AIE polymers for a variety of applications^32–35^. On the contrary, this approach has been surprisingly overlooked in the AIE-based LSC literature so far, with the only relevant example being the synthesis of luminescent poly(methyl methacrylate) (PMMA) polymers by AIE-molecule-initiated atom-transfer radical polymerization (ATRP), and their application in LSC devices with excellent light-harvesting response enabled by the homogeneously distributed AIE*gen* molecules^36^. In particular, the use of AIE-based monomeric structures to obtain macromolecular networks through controlled polymerization techniques for LSC systems has not been demonstrated in the literature to date, despite its great potential in enhancing the photonic response of LSCs via tunable control of luminophore arrangement and luminescent emission.

To bridge this gap, in this work, we develop a series of luminescent copolymeric structures based on a new monomeric AIE*gen* (2-(methacryloyloxy)ethyl 4-(1,2,2-triphenylvinyl)benzoate), **TPEMA**) co-polymerized in the presence of methyl methacrylate (MMA) in different relative proportions. To assess the effect of synthetic strategy on the chemical, physical, thermal, optical and photonic response of such systems, two different polymerization techniques are used, namely free-radical (FR) polymerization and reversible addition-fragmentation chain transfer (RAFT) polymerization. The resulting AIE-based copolymers are employed as luminescent layers in LSC devices, and their photonic and PV performance is systematically investigated, highlighting relevant structure-property relationships.

## Results and discussion

### Synthesis and photophysical characterization of TPEMA

The synthesis of **TPEMA** involved a Steglich esterification reaction between 4-(1,2,2-triphenylvinyl)benzoic acid (**TPE–COOH**) and 2-hydroxyethyl methacrylate (HEMA), conducted in the presence of dichloromethane (DCM) as a solvent, diisopropylcarbodiimide (DIC) as a coupling agent, and 4-dimethylaminopyridine (DMAP) as a catalyst under inert conditions.

**Fig. 1:**
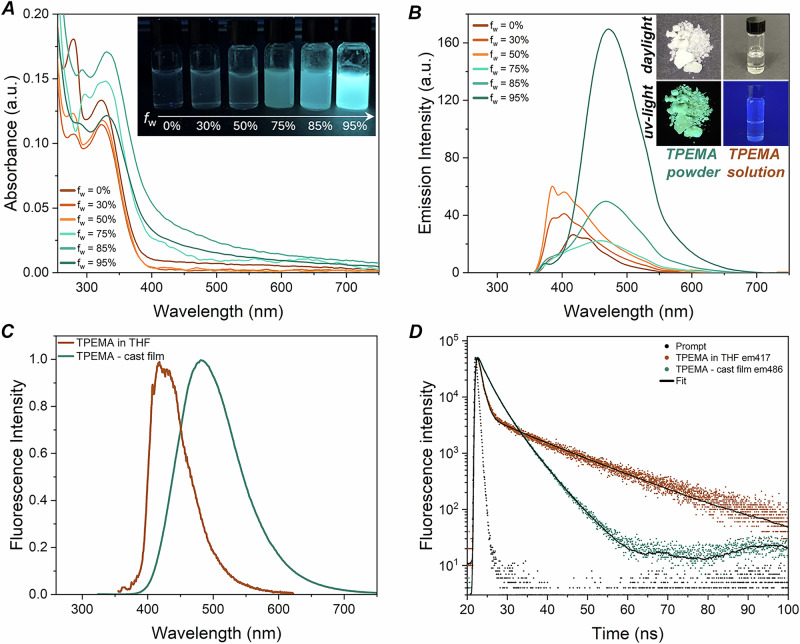
Photophysical characterization of TPEMA monomer. **A** UV absorption and **B** photoluminescence emission behavior of **TPEMA** in THF–H_2_O mixtures (10^−6^ M) with different volume fractions of water (*f*_w_ = 0%, 25%, 35%, 50%, 75%, 95%). In the inset: **A** photographic image of the solutions under a TLC UV lamp (@ 366 nm) and **B** photographs of **TPEMA** monomer at the solid state as a powder (left) and in THF solution (right), both observed under daylight (top) and UV light (bottom). **C** Normalized PL spectra of **TPEMA** in THF diluted solution (10^−6^ M) and as a cast film, **D** emission decays (scatter plots): **TPEMA** in THF at 417 nm with bi-exponential fit (solid line) and **TPEMA** as cast film at 486 nm with three-exponential fit (solid line).

To investigate the absorption/emission response of **TPEMA**, its photophysical behavior was studied in THF–H_2_O mixtures with varying water content. UV absorption and PL emission spectra were recorded for **TPEMA** solutions with volume fractions of water (*f*_w_) of 0%, 30%, 50%, 75%, 85%, 95% (as shown in Fig. 1). In this mixed-solvent system, THF acts as a good solvent for **TPEMA**, while water serves as a poor solvent, inducing aggregation at higher water fractions. This experimental setup allows for the qualitative observation of the AIE effect, where emission intensity is expected to increase as the water content rises, promoting molecular aggregation (visible in the inset of Fig. 1A). At lower volume fractions of water, **TPEMA** exhibits a primary absorption peak at ~323 nm, accompanied by a secondary peak at 280 nm. As *f*_w_ increases to 75% and beyond, the main peak becomes more pronounced, with a slight bathochromic shift observed.

With increasing volume fraction of water, the PL emission spectra undergo a progressive change in their profile, with the disappearance of the bands at shorter wavelengths (*λ* < 450 nm) accompanied by the appearance of a strong emission band centered at 470 nm for *f*_w_ ≥ 75%. This twofold response, which correlates positively with increasing water content, may be associated with a change occurring in the packing mode of **TPEMA** molecules in the aggregates^37^. Encouragingly, the main emission signal at 470 nm is found to increase in intensity with increasing *f*_w_, consistent with the AIE (or with the aggregation-induced emission enhancement—AIEE)^38,39^ phenomenon.

The steady-state and time-resolved photophysical properties of **TPEMA** were investigated in both solution and solid state. In a highly diluted THF solution, the monomer showed a low PLQY of 2.3%, which is expected due to the free molecular motion allowing non-radiative relaxation pathways to dominate, leading to weak fluorescence. In contrast, when aggregated in the solid state, the PLQY increased significantly to 36%, reflecting the characteristic behavior of AIE/AIEE compounds where molecular motion is restricted, enhancing radiative decay and fluorescence (Supplementary Table 6). Additionally, a shift in the emission wavelength was observed from 417 nm in solution to 486 nm in the solid state, with faster initial decay dynamics in solution evidencing the presence of non-radiative recombination processes (Fig. 1C). This red-shift can be attributed to molecular packing and intermolecular interactions in the solid state, which stabilize the excited state, reducing the energy gap and leading to a lower-energy emission. Altogether, these results clearly demonstrate typical AIE/AIEE behavior, where aggregation reduces non-radiative decays and enhances fluorescence efficiency.

### Copolymerization of the TPEMA monomer with MMA via free radical and RAFT polymerization

**Fig. 2:**
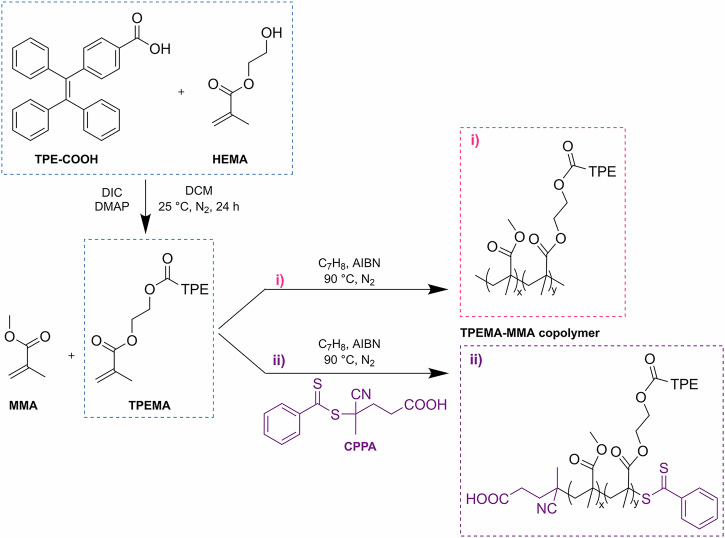
Synthesis of TPEMA and random copolymers via free-radical and RAFT polymerization. Reaction schemes of **A** **TPEMA** monomer and **B** **TPEMA–MMA** random copolymers via (i) free radical polymerization and (ii) RAFT polymerization.

**TPEMA** was subjected to copolymerization with MMA at various molar ratios (1:99, 10:90, 20:80, 50:50, 75:25), employing two distinct radical polymerization methods: free-radical (FR) polymerization and controlled RAFT polymerization (for the name assigned to each copolymer synthesized, refer to Table 1). In FR copolymerization, **TPEMA** and **MMA** were polymerized in dry toluene with AIBN as the initiator at 90 °C. RAFT polymerization followed a similar process, but with the addition of a RAFT agent (4-cyano-4-(phenylcarbonothioylthio) pentanoic acid, **CPPA**). The conversion during copolymer preparation was monitored using ¹H-NMR. The synthetic approach used to obtain the **TPEMA** monomer and its copolymers with MMA, following the two different synthetic routes, is reported in Fig. 2 and described in detail in the “Methods” section.

**Table 1:** Compositional and physical properties of **TPEMA–MMA** copolymers prepared by FR and RAFT polymerization

Sample	Feed composition (%) TPEMA/MMA	Observed composition (%) TPEMA/MMA	Yield^a^ (%)	*M*_n_^b^ (kg/mol)	*Đ* ^b^	*T*_g,exp_^c^ (°C)	*T*_g,theo_^d^ (°C)
FR1-99	1/99	1/99	59	27.7	1.82	81	101
FR10-90	10/90	11/89	49	18.3	1.91	126	107
FR20-80	20/80	18/82	24	11.5	1.66	128	111
FR50-50	50/50	50/50	64	8.5	1.94	125	118
FR75-25	75/25	72/28	56	11.5	1.76	124	121
RAFT1-99	1/99	1/99	56	11.1	1.15	84	101
RAFT10-90	10/90	12/88	42	8.7	1.21	106	107
RAFT20-80	20/80	24/76	36	11.8	1.21	112	111
RAFT50-50	50/50	51/49	52	15.5	1.18	114	118
RAFT75-25	75/25	71/29	49	14.6	1.21	121	121

^a^The yield of the reaction is calculated as the ratio between the mass of the copolymer obtained at the end of the synthesis and the sum of the masses of the reagents in the feed (considering also the RAFT agent in the case of RAFT polymerizations).

^b^Determined through GPC measurements.

^c^Determined through DSC measurements.

^d^*T*_g_ values predicted using the Fox–Flory equation^47^.

Feed compositions (theoretical), observed compositions (real) from ^1^H-NMR spectra and physical properties of **TPEMA**–**MMA** copolymers obtained via random FR (top) and RAFT (bottom) polymerization: number-average molecular weight (*M*_n_); dispersity (*Đ*); *T*_g_ values obtained experimentally (*T*_g,exp_) and theoretically (*T*_g,theo_).

**Fig. 3:**
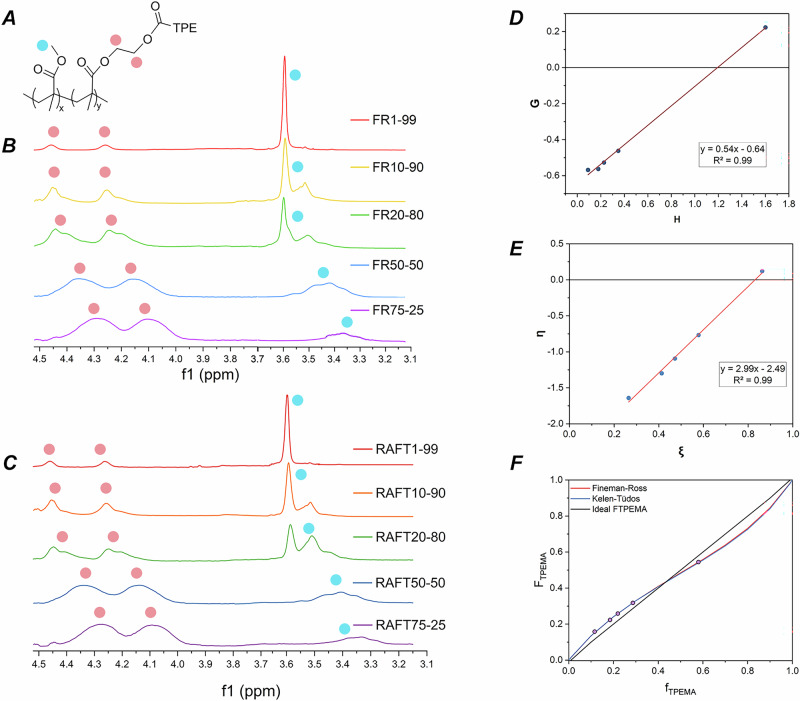
Reactivity study of copolymers TPEMA–MMA with increasing content of TPEMA. **A** Copolymer structure. **B** Stacked ^1^H-NMR spectra of the final **TPEMA–MMA** copolymers obtained via FR and **C** RAFT polymerization, in pink dots are signed the **TPEMA** ethylene CH_2_–CH_2_ protons, while in light blue dots the **MMA** methoxy O-CH_3_. **D** Trend line fitting of the experimental data, according to Fineman–Ross method. Fitting equation: *y* = 0.54*x*−0.64, with *R*² = 0.99. **E** Trend line fitting of the experimental data, according to the Kelen–Tüdos method. Fitting equation: *y* = 2.99*x*–2.49, with *R*² = 0.99. **F** Diagram of monomer composition according to the Mayo–Lewis equation, including the trend of the ideal behavior (diagonal). The experimental points obtained by low-conversion polymerizations at different inlet compositions are also reported.

The output molar composition of the synthesized random copolymers (both FR polymerization and RAFT polymerization) was assessed through ^1^H-NMR spectroscopy and was shown to closely match the feed composition (Table 1), indicating good control over the synthetic process. Interestingly, the analysis of the proton spectra for the different **TPEMA–MMA** copolymers provided additional insights into the distinct arrangement of the monomeric units within the copolymeric structure. Notably, variations in the **TPEMA**:**MMA** molar ratio could be correlated with changes in the relative intensity of the relevant signals observed at 3.65–3.20 ppm (corresponding to the methoxy group of the MMA unit in the polymer—Fig. 3B, C blue highlight) and at 4.50–3.95 ppm (associated with the ethylene bridge of the **TPEMA**—Fig. 3A, B red highlight). These spectral changes suggest modifications in the sequence and composition of the monomers within the copolymer, influenced by the different reactivities of **TPEMA** and **MMA**. As a result, the microstructure of the copolymer may be affected, altering the interatomic interactions at a local level and impacting the NMR spectral response. Indeed, different signals were found to progressively appear, disappear or shift by varying the relative **TPEMA**:**MMA** molar concentration, consistent with the expected disposition and abundance of **MMA** and **TPEMA** units within the copolymer. More specifically, by increasing the **TPEMA** content, the sharp **MMA** peak at 3.55 ppm modifies into a multiplex of peaks at a slightly lower ppm value. The shifts in the four main peaks observed in the NMR spectra within the range of 3.5–3.2 ppm, together with the corresponding monomer sequences to be expected, are depicted in Supplementary Fig. 7 (sequences a–d), offering a nuanced understanding of the copolymeric composition based on these spectral variations.

To deepen our understanding of the copolymerization process, a reactivity study was performed based on the linearization of the Mayo–Lewis equation^40^ according to the Fineman–Ross method^41^, and Kelen–Tüdos method^42,43^. Copolymerization reactions were conducted at various feed compositions and maintained at low conversions (<10%, see more details in Section 2.2, Supplementary Information). In particular, the reactivity ratios calculated from the slope and the *H*-axis intercept of the Fineman–Ross *G*–*H* plot (Fig. 3D) were found to be *r*_TPEMA_ = 0.54 and *r*_MMA_ = 0.64.

Further enhancing the analysis, the Kelen–Tüdos method was also employed (Fig. 3E), known for its utility in handling datasets with limited experimental data or for achieving higher precision in estimating reactivity ratios. This method yielded reactivity ratios of *r*_TPEMA_ = 0.51 and *r*_MMA_ = 0.63 (see more details in Section 2.2, Supplementary Information). The close values of *r*_TPEMA_ and *r*_MMA_, as determined by both the Fineman–Ross and Kelen–Tüdos methods, suggest a relatively balanced reactivity between the two monomers. In accordance with the terminal kinetic model, a preferential tendency for the two co-monomers to undergo cross-propagation rather than homo-propagation during the polymerization process is expected, since both values of reactivity ratios are lower than 1. As the content of **TPEMA** increases in the copolymer, the potential for steric hindrance could also increase, which may affect its ability to incorporate into the polymer chain. This steric hindrance can be generally associated with the bulkier structure of **TPEMA**, which might slow down the growth of the active kinetic chain, especially at higher concentrations of _**TPEMA**_. Thus, while the overall copolymerization leads to a nearly random distribution of monomers, the presence of increasing amounts of **TPEMA** could introduce slight variations in this pattern due to its steric effects^40^. These variations could result in a less uniform reactivity and minor deviations from an ideal random copolymer structure as **TPEMA** content increases, potentially influencing the physical properties of the polymer (Fig. 3F).

All the copolymers obtained from both FR and RAFT polymerization processes were characterized in detail using gel permeation chromatography (GPC), differential scanning calorimetry (DSC), thermogravimetric analysis (TGA), and optical transmittance (%*T*). As expected, RAFT polymerization allows a higher control over dispersity (*Đ*), with values falling in the range of 1.15–1.21, as reported in Table 1, whereas *Đ* values ranging from 1.66 to 1.94 were obtained via the FR process. Interestingly, DSC analysis revealed an increase in the *T*_g_ upon increasing **TPEMA** content irrespective of the synthetic method, with values in the 80–130 °C range. This result suggests reduced molecular mobility due to the bulkier structure of **TPEMA** compared to MMA, leading to enhanced rigidity, decreased free volume and a more extended glassy behavior of the material^44–46^. Notably, in contrast to copolymers synthesized via FR polymerization, the *T*_g_ of RAFT copolymers increased more gradually and consistently with the molar content of **TPEMA**, aligning well with the values theoretically predicted by the Fox–Flory equation (*T*_g,theo_ in Table 1; see Supplementary Eq. (11))^47^. This finding highlights the benefits of using RAFT polymerization for controlling not only the distribution of polymeric chain lengths but also the physical properties of the obtained copolymers. Finally, TGA measurements under a nitrogen atmosphere revealed excellent thermal stability up to at least 150 °C for all synthesized materials, which was found to be increasing for higher **TPEMA** molar content. Such a feature is consistently more evident in the case of the RAFT series (Section 2.7, Supplementary Information).

### Photophysical properties of AIE*gen*-containing copolymers

**Fig. 4:**
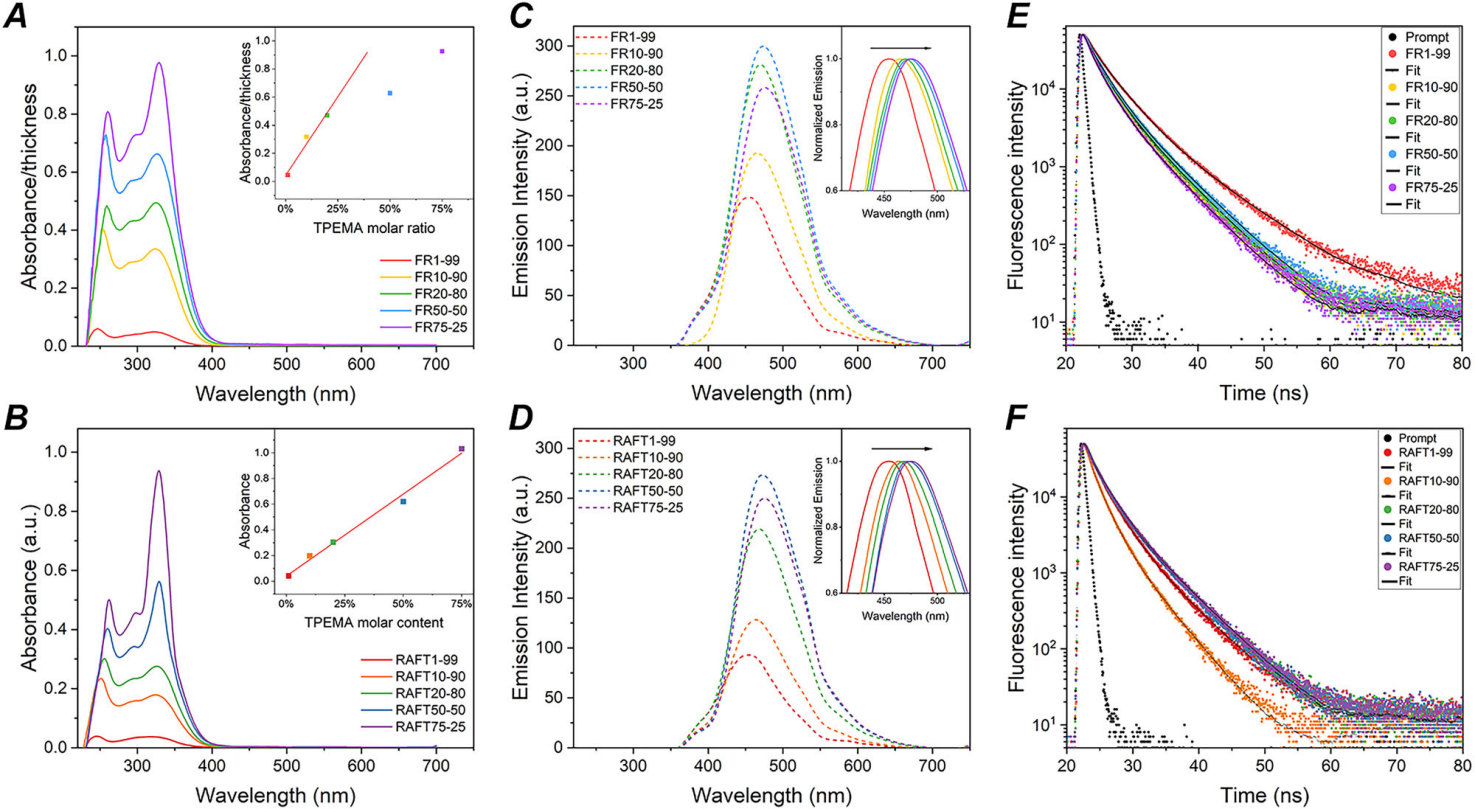
Photophysical characterization of TPEMA–MMA copolymers. **A** UV–Vis absorbance spectra for copolymers obtained via FR polymerization; inset: normalized absorption peak (*λ*_max1_ = 320–330 nm) as a function of **TPEMA** molar ratio. **B** UV–Vis absorbance for copolymers via RAFT polymerization; inset: absorbance vs. **TPEMA** molar ratio. PL spectra for (**C**) FR synthesized copolymers and **D** RAFT synthesized copolymers; inset: Normalized emission. Emission decays (scatter plots) for (**E**) FR copolymers and **F** RAFT copolymers.

The solid-state absorption and emission response of **TPEMA–MMA** copolymers were recorded in thin-film configuration (Fig. 4). All the systems displayed a main absorption peak at *λ*_max1_ = 320–330 nm and a secondary peak at a lower wavelength (*λ*_max2_ = 245–260 nm), separated by a shoulder. An enhancement in the absorption intensity was observed with increasing **TPEMA** molar content. For the copolymers synthesized through a controlled RAFT polymerization, a linear increase in absorbance with **TPEMA** concentration was observed, consistent with the Lambert–Beer law (inset of Fig. 4B). On the contrary, the FR series exhibited deviations from this linear trend (Fig. 4A) likely due to inhomogeneity in the spatial distribution of the AIE/AIEE species within the macromolecular sequence, resulting from the poorer control over the molecular weight distribution achieved with FR polymerization vs. RAFT polymerization. This also contributed to less uniform coating thicknesses (see Supplementary Table 5), potentially causing scattering phenomena within the film and random packing direction of the TPE units in the polymeric chain. To account for differences in film thickness, absorption values were normalized by the thickness and plotted (Fig. 4A). Even after the thickness correction, the absorbance increased with deviations from the Lambert–Beer law for **TPEMA** molar contents above 20%.

The steady-state fluorescence emission spectra for both FR and RAFT **TPEMA–MMA** copolymers are characterized by an intense broad band spanning from 350 to 700 nm, with an emission peak found to red-shift from 454 to 476 nm for increasing **TPEMA** molar content (Fig. 4C, D). Such bathochromic shift, attributable to increased aggregation of the **TPEMA** moieties and to extended conjugation length occurring with higher fractions of **TPEMA**, is found to correlate linearly with absorption only for the RAFT series (Supplementary Fig. 29). This behavior may suggest that the RAFT polymerization method ensures an ideal spatial distribution of **TPEMA** within the copolymer sequence, which favors consistent fluorescent and AIE/AIEE behavior. As expected for AIE/AIEE materials, the emission intensity of the copolymers was found to increase with **TPEMA** content (i.e., the AIE*gen* species), reaching a maximum for the **TPEMA:MMA** = 50:50 composition in both FR and RAFT series. On the contrary, higher **TPEMA** molar contents (**TPEMA:MMA** = 75:25) led to a slight decrease in the emission intensity, which can be associated with ACQ phenomena. Indeed, as reported in several studies, this effect can still occur in AIE systems under certain conditions, as a result of other physical phenomena causing such PL reduction^36^.

To gain more detailed information on the photophysical behavior of the **TPEMA–MMA** copolymers, the PLQY and fluorescence decay dynamics of spin-coated films were evaluated (Table 2, Fig. 4E, F and Supplementary Information, Section 3). Both the copolymers of the RAFT and FR series exhibited a similar trend in PLQY and average decay times *τ* (see Table 2). In particular, both FR and RAFT series exhibit PLQY values comparable to those obtained from **TPEMA** monomer in the solid state (PLQY = 36%, Supplementary Table 6), proving that in the synthesized copolymer films, the AIE/AIEE behavior of the parent **TPEMA** moiety is well preserved. When comparing the results from the two different series, an increasing trend is observed in PLQY values for the RAFT copolymers for increasing **TPEMA** contents, from 23% in the most **TPEMA**-diluted system (RAFT1-99) to 41% in the most **TPEMA**-concentrated system (RAFT75-25). On the other hand, no clear PLQY trend is found in the FR series at varying **TPEMA** concentration. This different behavior may be correlated with the more regular macromolecular chain length distribution (viz., small *Đ*) achievable through RAFT polymerization vs. FR polymerization, which is expected to favor better control of both intramolecular and intermolecular interactions between AIE/AIEE moieties, providing more consistent photonic response (as will be discussed in the following section).

**Table 2:** Photophysical properties of **TPEMA–MMA** copolymer films

Sample	${\lambda }_{{{\rm{a}}}{{\rm{b}}}{{\rm{s}}}}^{max}$ (nm)		${{{\boldsymbol{\lambda }}}}_{{{\boldsymbol{em}}}}^{{{max }}}$ (nm)	PLQY (%)	*τ* (ns)^a^
	*λ*_max1_	*λ*_max2_			
FR1-99	323	247	455	52	2.65
FR10-90	325	254	465	47	1.75
FR20-80	326	259	470	48	1.79
FR50-50	326	258	474	40	1.78
FR75-25	329	261	476	38	1.56
RAFT1-99	319	245	454	23	1.52
RAFT10-90	325	252	464	20	1.01
RAFT20-80	326	256	468	38	1.56
RAFT50-50	329	260	472	38	1.49
RAFT75-25	330	262	475	41	1.59

^a^From three-exponential fits (see Supplementary Information, Section 3).

Photophysical properties of the **TPEMA–MMA** films: Absorption (${\lambda }_{{{\rm{a}}}{{\rm{b}}}{{\rm{s}}}}^{max}$) and fluorescence maximum positions (${\lambda }_{{{\rm{e}}}{{\rm{m}}}}^{max}$); Stokes shift; PLQY; Average lifetime decay (*τ*).

In addition to steady-state properties, the photostability of representative copolymers (FR50-50 and RAFT50-50) was also evaluated under continuous AM 1.5 G solar irradiation (100 mW cm⁻²). Both systems exhibited a gradual reduction in PL intensity (ca. 40–45%) with no appreciable spectral shifts, indicating that the presence of RAFT end-groups does not significantly alter the degradation rate. UV–vis, PL, and FTIR analyses confirmed only minor chemical changes after prolonged irradiation, consistent with good backbone stability (Supplementary Figs. 27 and 28).

### Optical and photovoltaic characterization of thin-film TPEMA–MMA LSC devices

**Fig. 5:**
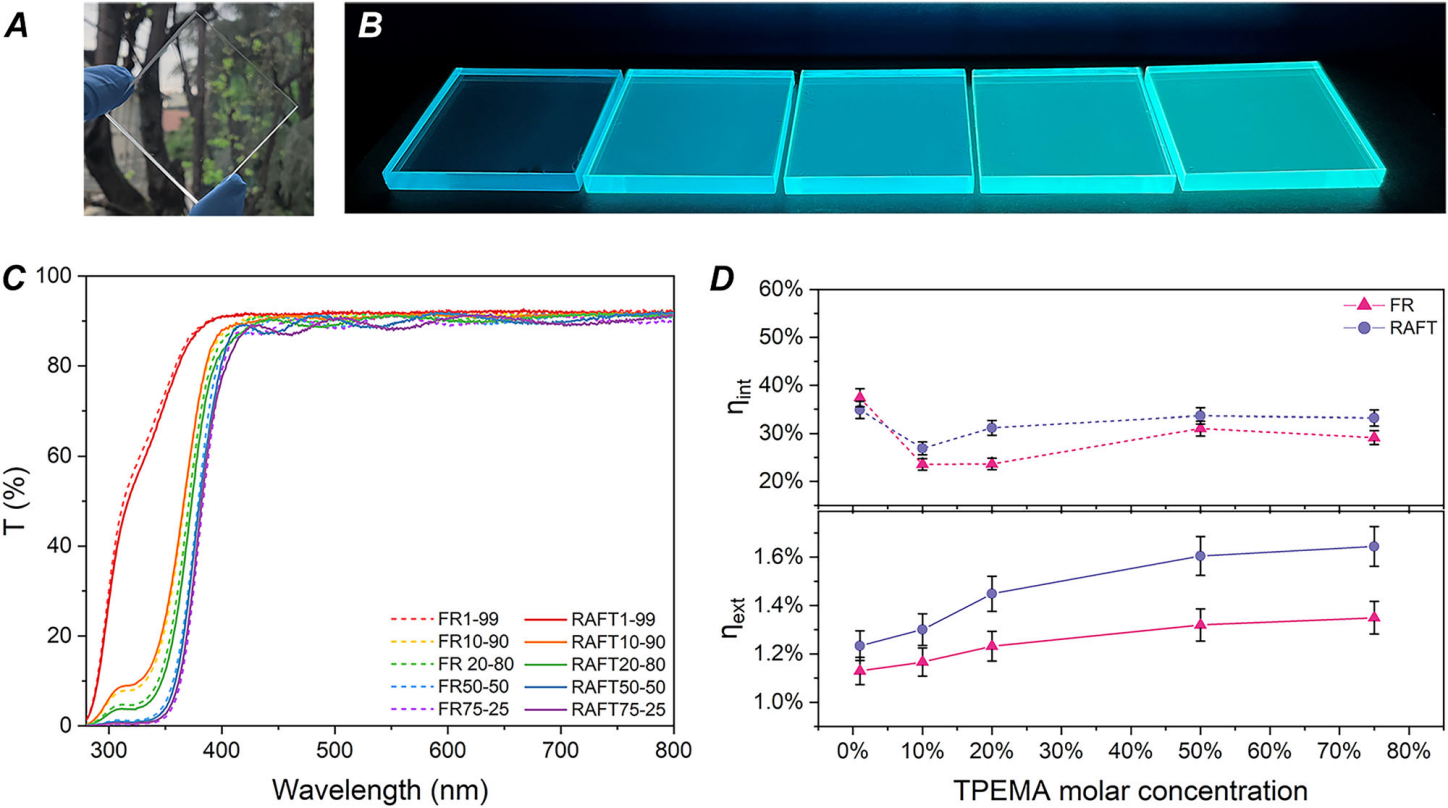
TPEMA/MMA LSCs and their photonic response. **A** Photographic image illustrating the transparent and colorless nature of the **TPEMA–MMA** LSCs. **B** Photographic image of **TPEMA–MMA** LSCs, RAFT series, under a TLC UV lamp (@ 366 nm) of the fabricated LSCs showing a red-shift (color change) in emission occurring with increasing **TPEMA** molar content inside the copolymers (from left to right). **C** Transmission spectra of **TPEMA–MMA** LSCs (both FR and RAFT series). **D** Internal (*η*_int_) and external (*η*_ext_) photon efficiencies of **TPEMA–MMA** LSCs.

Based on the findings discussed thus far, thin-film LSC devices based on **TPEMA–MMA** copolymers were fabricated on 5.0 × 5.0 × 0.6 cm^3^ N-BK7 glass slabs. The esthetic quality of the obtained films was assessed through chromaticity and transmittance measurements. More specifically, to gauge the visual appearance of the LSCs under standard illumination conditions (AM 1.5 G), the conventional Commission Internationale de l’Eclairage (CIE) 1931 *xy* coordinates system was employed (Supplementary Fig. 33). All systems converged in one single point (*x*, *y* = 0.33, 0.34) close to the white-neutral central region, demonstrating the colorless and transparent feature of the fabricated devices (Fig. 5A). In addition, UV light exposure evidenced a red-shifted emission of the films - visible as slight color change from blue to greenish—for increasing **TPEMA** molar content in the copolymer (Fig. 5B), in line with previous discussion (Fig. 4).

To quantify the visible transparency of the LSC systems as perceived by the human eye’s photopic response, average visible transmittance (AVT) analyses were conducted through the evaluation of the spectrally resolved transmittance (T%) of the devices by means of UV–Vis spectrophotometry (see Supplementary Eq. (17)). High *T*% values (AVT ~ 90%) were recorded for all LSC devices (Fig. 5C and Supplementary Table 10), confirming their potential as high-transparency and colorless solar energy devices^3^. Furthermore, morphological analysis of the copolymer films (see Supplementary Information, Section 3.2, Supplementary Fig. 26) revealed uniform, featureless surfaces with no evidence of phase separation or aggregation, confirming that the incorporation of **TPEMA** into the polymeric matrix yields homogeneous luminescent layers suitable for LSC devices.

To assess the performance of thin-film **TPEMA–MMA** LSCs as photonic devices, each system was irradiated with AM1.5 G solar simulated light (100 mW cm^−2^), and two key parameters were calculated, namely the external (*η*_ext_) and the internal (*η*_int_) photon efficiencies (Supplementary Eqs. (14) and (15)^6^. As shown in Fig. 5D, both FR and RAFT series were found to follow a similar trend for *η*_ext_ and *η*_int_. More specifically, the *η*_ext_ increased with the **TPEMA** molar content in the copolymer, as a result of the greater number of emitted photons resulting from the higher relative concentration of AIE*gen* moieties in the system. Conversely, a relatively constant *η*_int_ was observed (∼33%) for copolymers incorporating higher concentrations of **TPEMA** (20–75 mol%). As expected, this trend agrees well with that observed for the PLQY at the same **TPEMA** contents. In comparing the two polymerization methods, the RAFT series exhibited notably higher *η*_ext_ values compared to the FR series (1.23–1.64% with RAFT polymerization vs. 1.13–1.35% with FR polymerization). Similarly, the RAFT series also demonstrated slightly higher and more consistent values for *η*_int_, with average values around ~33% (detailed measurements are reported in Section 4.2 in the Supplementary Information). These results further corroborate the benefits of using RAFT polymerization to obtain AIE-based LSC devices with superior photonic response, as a result of the more refined control over the macromolecular characteristics of such systems.

Finally, the PV characteristics of the obtained LSC devices were recorded, and their power conversion efficiency (*η*_dev_) was calculated. LSC assemblies were fabricated by connecting the photoactive regions of two mc-Si PV modules to opposite edges of the glass substrate. Photocurrent measurements on the LSCs were performed under AM1.5 G simulated sunlight irradiation (100 mW cm⁻²), using a black absorbing background and black tape on the uncovered edges to prevent efficiency overestimation due to double crossing of internally reflected photons. Based on the results of the previous optical and photonic characterization performed on both FR and RAFT-based copolymers, the **TPEMA:MMA** = 50:50 molar composition was selected as a case study for the measurement of PV performance of the resulting LSCs. Both FR50-50 and RAFT50-50 systems were analyzed to allow fair comparison of the outputs. As a result, maximum *η*_dev_ values of 0.23% and 0.29% were obtained for the FR and RAFT series, respectively (details on calculation methods, experimental features and characteristic PV data can be found in Section 4.3 in the Supplementary Information). To facilitate the comparison with benchmark devices with different AVT and optical density, and to provide an estimation of the usability of these systems as colorless LSCs, their light utilization efficiency (LUE) was calculated (see Supplementary Eq. (19) and Supplementary Table 10), which represents a comprehensive indicator of both transparency and PV efficiency (LUE = AVT·*η*_dev_)^48^. The target LSC devices based on FR50-50 and RAFT50-50 copolymers exhibited LUE values of 0.21% (AVT ~ 89%) and 0.25% (AVT ~ 86%), respectively.

## Conclusion

This study demonstrates the effective integration of monomeric AIE*gens* (**TPEMA**) into polymeric backbones for LSCs using free radical (FR) polymerization and reversible addition-fragmentation chain transfer (RAFT) polymerization. The RAFT method allowed for enhanced control over the molecular weight distribution of the obtained copolymers and produced systems with more consistent *T*_g_ values and improved thermal stability, which were found to increase with the concentration of the AIE moiety within the copolymers. Notably, their PL emission intensity increased significantly with the **TPEMA** molar fraction, achieving a maximum at the 50:50 molar composition. LSC devices incorporating the AIE-based copolymers obtained through FR or RAFT polymerization exhibited AVT values around 90%, indicating high visible transparency. In addition, RAFT-based LSCs were found to yield higher and more consistent photonic and photovoltaic performance than FR-based systems, with *η*_ext_ values between 1.23% and 1.64%, *η*_int_ ≈ 33% and *η*_dev_ = 0.29%. As a result, RAFT polymerization proved to be a more effective approach for developing luminescent copolymers for LSCs with AIE/AIEE behavior, providing tailored macromolecular control for improved device response. This work demonstrates a promising approach for enhanced LSC response through the synthetic control of the macromolecular network and the strategic integration of AIE*gens* into luminescent polymer matrices, and paves the way for the development of advanced AIE-active macromolecules for solar energy technologies.

## Methods

### Materials

4-(1,2,2-Triphenylvinyl)benzoic Acid (TPE–COOH) was purchased from Activate Scientific and used with no further purification. 4-Dimethylaminopyridine (DMAP), dichloromethane (DCM), Diisopropylcarbodiimide (DIC), 4-Cyano-4-(phenylcarbonothioylthio)pentanoic acid (CPPA), methanol, tetrahydrofuran (THF), chloroform, CDCl_3_, hexane, ethyl acetate were purchased from Sigma Aldrich and used with no further purification. 2-Hydroxyethyl methacrylate (HEMA), methyl methacrylate (MMA) and toluene were purchased from Sigma Aldrich. α,α′-Azobisisobutyronitrile (AIBN) was purchased from Fluka. Monocrystalline high-efficiency silicon solar cells were provided by IXYS (IXOLAR SolarBIT KXOB25-12X1L active area = 2.2 × 0.6 cm^2^, *V*_OC_ = 0.67 ± 0.01 V, *J*_SC_ = 53.60 ± 0.42 mA cm^−2^, FF = 69.4 ± 0.3%, power conversion efficiency (PCE) = 24.69 ± 0.23%).

### Purification methods

Prior to functionalization and polymerization, 2-hydroxyethyl methacrylate (HEMA) and methyl methacrylate (MMA) were purified to remove inhibitors. HEMA was passed through a short column of activated basic alumina (aluminum oxide, Brockmann I) while MMA was washed 3 times with a 1 M aqueous sodium hydroxide (NaOH) solution, followed by separation of the organic layer, drying over anhydrous magnesium sulfate, and filtration. Both purified monomers were stored under refrigeration (4 °C) in sealed containers to prevent self-polymerization prior to use.

α,α′-Azobisisobutyronitrile (AIBN) was purified by recrystallization prior to use. AIBN was added to a conical flask containing methanol and stirred while gently heating the mixture to 50 °C to ensure complete dissolution. The resulting solution was allowed to cool to room temperature, followed by further cooling in an ice bath to initiate crystal formation. The flask was then placed in a freezer for 48 h to promote slow crystal growth. The recrystallized AIBN was collected by vacuum filtration and dried under reduced pressure. This procedure was repeated twice to ensure high purity.

Dry toluene was obtained by distillation from a suspension of calcium hydride (CaH₂) under an argon atmosphere. The dried solvent was stored over 3 Å molecular sieves in a sealed container until use. Analytical thin-layer chromatography was performed on silica gel, chromophore-loaded, commercially available plates.

### Synthetic procedures

Synthesis of 2-(methacryloyloxy)ethyl 4-(1,2,2-triphenylvinyl)benzoate) (**TPEMA**) (compound **2**). A flame-dried, nitrogen purged 50 mL two-neck flask was charged with a solution of TPECOOH (752 mg, 2 mmol) in dry DCM (20 mL) with magnetic stirring. The solution was then cooled at 0 °C and DIC (340 μL,2.20 mmol) was added. and the mixture was stirred for 10–15 min; DMAP (122 mg, 0.99 μmol) and HEMA (275 μL, 2 mmol) were subsequently added. The reaction mixture was allowed to warm to room temperature and stirring was continued for 24–48 h. The reaction mixture was extracted with an ammonium chloride (NH_4_Cl) aqueous solution, brine (3×), and then dried (Na_2_SO_4_). The product was purified by column chromatography (SiO_2_; hexane:ethyl acetate 25:1) to yield a light yellow/white solid, (650 mg, 67%). ^1^H-NMR (400 MHz, CDCl_3_) *δ* = 7.77 (d, *J* = 8.4 Hz, 2H), 7.11 (td, *J* = 4.0, 3.5, 2.0 Hz, 12H), 7.02 (dd, *J* = 5.6, 2.1 Hz, 5H), 6.12 (s, 1H), 5.58 (s, 1H), 4.55–4.48 (m, 2H), 4.48 – 4.40 (m, 2H), 1.94 (s, 3H)^13^.C-NMR (101 MHz, CDCl_3_) *δ* = 149.23, 143.24, 142.70, 131.51, 131.40, 129.25, 128.00, 127.86, 127.04, 126.89, 126.19, 62.67, 62.60, 18.41. ESI-MS *m*/*z*: [M + 1] = 489. Anal. Calcd. for C_33_H_28_O_4_: C, 78.43; H, 5.83. Found: C, 78.39; H, 5.77.

### Copolymerization procedures

Synthesis of poly(MMA-co-TPEMA). A solution of the monomers (**TPEMA** and **MMA**) in dry toluene was loaded into a flame-dried, nitrogen purged 4 mL vial and nitrogen bubbling was carried out for about 10 min to eliminate the air present in the solution. It was then added under nitrogen to a solution of the initiator (AIBN) and eventually the RAFT agent (CPPA) in dry toluene. The reaction vial was positioned on a magnetic stirrer, in an oil bath at 90 °C, allowing the polymerization to proceed. Finally, the resulting copolymer was cooled to room temperature, and the reaction was quenched by air. The final product was obtained by first precipitating it in cold methanol, then filtering it under vacuum, and finally drying it in a vacuum dryer.

In the case of FR, the mass of AIBN employed was equal to 1 wt% of the total mass of the monomers (**TPEMA** + **MMA**); in the case of RAFT polymerization, the ratio [CPPA]:[AIBN] was kept constant to 1:0.3 for all the experiments, while the imposed ratio of monomers to CPPA was equal to 100:1.

^1^H-NMR (400 MHz, CDCl_3_) *δ* = 7.76 (d, 2H), 7.08 (m, 12H), 7.00 (m, 5H), 4.50–3.95 (m, 4H), 3.65–3.20 (m, 3H).

### Spectral characterization

^1^H NMR (400 MHz) and ^13^C NMR (100 MHz) spectra were recorded on a Bruker Avance 400 using deuterated chloroform as solvent. Chemical shifts (δ) are reported in parts per million (ppm) relative to the residual solvent peak (*δ*_Η_ = 7.26 ppm and *δ*_C_ = 77.16 ppm for CDCl₃). All spectra were acquired at room temperature with 32 scans and a relaxation delay of 5 s. Spectra were processed and analyzed using MestReNova software (Mestrelab Research).

Mass spectra of pure compounds were recorded using an electrospray ionization (ESI) ThermoFisher Technologies mass spectrometer with a quadrupole as mass analyzer. Sample was prepared by dissolving compound **2** in DCM (20 ppm) with a drop of TFA.

### Gel permeation chromatography (GPC) analysis

Gel permeation chromatography (GPC) was performed on a Waters instrument (mobile phase, THF stabilized with BHT (2,6-di-t-butyl-4-methylphenol); flow rate, 1 mL min^−1^, at 40 °C) consisting of two universal columns in series (Styragel 4E and 5E) for separations ranging from 500 to 10^6^ Da and a refractive index detector. Samples obtained via FR or RAFT polymerization were dissolved in THF at a concentration in the range 4–10 mg mL^−1^, prefiltered using 0.45 µm polytetrafluoroethylene filters and then directly injected. Molecular weight distribution data (*M*_w_, *M*_n_ and *Ð*) have been obtained through elaboration with the software Breeze, using 12 low polydispersity polystyrene standards for the calibration curve (Fluka kit).

### Thermal analysis

Differential scanning calorimetry (DSC) analyses were performed with a DSC 823e Mettler-Toledo. Approximately 10 mg of the sample was weighed into an aluminum pan, which was then hermetically sealed. The thermal cycle applied consisted of an initial heating from 25 to 100 °C, followed by cooling to −20 °C, and a second heating step from −20 to 200 °C. All heating and cooling ramps were performed at a rate of 20 °C min^−1^ under a nitrogen atmosphere. Thermogravimetric analyses (TGA) were conducted with a Q500 thermogravimetric analyzer. Approximately 10 mg of sample was placed in a platinum pan and heated from 25 to 600 °C at a constant rate of 10 °C min^−1^ under nitrogen.

### Photophysical characterization

UV–visible absorption and transmission spectra were recorded using a Thermo Scientific Evolution 600 UV–vis spectrophotometer. The AIE behavior of compound 2 was evaluated by preparing a stock solution in tetrahydrofuran (THF). Aliquots of 10 μL from this stock were added to water–THF mixtures to a total volume of 2 mL, yielding various water fractions (*f*_w_) for solution at a concentration of 1 × 10^−6^ M. The solutions were analyzed in quartz cuvettes, and for each measurement, a background spectrum of the corresponding solvent mixture was recorded for baseline correction. The same procedure was followed for polymer solutions of FR50-50 and RAFT50-50.

For thin films and LSC devices, UV–vis absorption spectra were collected by placing the samples directly in the spectrophotometer beam path. Background spectra were obtained using clean optical glass substrates to isolate the absorption signal of the active materials. For transmission measurements and the calculation of AVT, background spectra were acquired in air, allowing for the contribution of the glass substrate.

Steady-state photoluminescence (PL) spectra were recorded on a Jasco FP-6600 spectrofluorometer. For thin films and LSCs, PL spectra were acquired in front-face configuration with the samples positioned at a 130° angle relative to the excitation beam. PLQY measurements of the films were performed by using a home-made integrating sphere according to the procedure reported elsewhere^49^ with a SPEX 270M monochromator equipped with a LN2-cooled charge-coupled device, by exciting with a monochromated 450 W Xe lamp at 370 nm. The spectra are corrected for the instrument response. Fluorescence time-resolved TCSPC measurements were performed with a NanoLog composed of an iH320 spectrograph equipped with a PPD-850 single photon detector module and a DeltaTime series DD-300 DeltaDiode and analyzed with the instrument software DAS6.

### Preparation of LSC in thin-film configuration and LSC-PV assembly

LSCs were fabricated in thin-film configuration starting from CHCl_3_ solutions of copolymer at 5 and 10 wt%. The obtained solution was spin-coated onto 5.0 × 5.0 × 0.6 cm^3^ N-BK7 high-optical-quality glass slabs using a Laurell WS-400BZ-6NPP/LITE instrument at 900 rpm, for 60 s. The thickness of the coatings was measured with a KLA Tencor P-17 Stylus profilometer. To obtain LSC-PV systems, four monocrystalline silicon solar cells connected in series were coupled to the devices by means of a thermosoftening EVA copolymer, so that two opposite edges of the glass substrates faced the photoactive area of two c-Si solar cells each. The other two edges were masked with black tape.

Internal and external photon efficiency (*η*_int_ and *η*_ext_, respectively) were assessed by illuminating the top face of the LSC using an Abet Technologies Sun 2000 solar simulator with an AM1.5 G filter (irradiance of 1000 W m^−1^) and by collecting the edge emitted photons of the LSC devices with a spectroradiometer (International Light Technologies ILT950) equipped with a cosine corrector. The spectra were recorded on a SpectrlLight III software.

Device efficiency measurements were performed using a Keithley 2612B source-measuring unit, connected in series with the circuit, which imposes voltage scans and measures the current output. An absorbing black background in contact with the LSC rear side and a black mask on the front face of the LSC system were used to prevent photon double-pass effects and direct illumination of the PV cells, respectively.

## Supplementary information

Supplemental material

Photovoltaic reporting

## Data Availability

The data supporting the findings of this study are included in the paper or in the Supplementary Information. All other information is available upon request from the corresponding author.
