# Supplementary material for: Tailoring aggregation-induced emission in luminescent solar concentrators through controlled polymerization
Source: Commun Chem. 2025 Oct 17;8:312. doi: 10.1038/s42004-025-01700-1 (PMC12534600; doi:10.1038/s42004-025-01700-1)
Supplement: Supplementary file 1 — Supplemental material [file 42004_2025_1700_MOESM1_ESM.pdf]

Supplementary Information

Tailoring aggregation-induced emission in luminescent solar concentrators through controlled polymerization

Elisavet Tatsi[a], Gaia Roberta Ragno[a], Andrea Nitti[b], Chiara Botta[c], Stefano Turri[a], Dario Pasini[b], Gianmarco Griffini[a]*

* Corresponding author E-mail: gianmarco.griffini@polimi.it

TABLE OF CONTENTS

1. Characterization of AIE luminogens 3
   1.1 NMR of TPEMA and TPE-COOH 3
   1.2 Differential Scanning Calorimetry (DSC) of TPE-COOH and TPEMA 6
   1.3 Thermogravimetric Analysis (TGA) of TPE-COOH and TPEMA 7
2. Characterization of polymers 8
   2.1 Synthesis and Characterization of pTPEMA homopolymer by FR and RAFT polymerizations 8
   2.2 Copolymers characterization 9
   2.3 NMR of RAFT agent, Methyl methacrylate and copolymers 11
   2.4 Reactivity Study 25
   2.5 Gel Permeation Chromatography (GPC) of copolymers 29
   2.6 Differential Scanning Calorimetry (DSC) of copolymers 30
   2.7 Thermogravimetric Analysis (TGA) of copolymers 32
   2.8 Profilometer Analysis 34
3. Photophysical Characterization 35
   3.1 Photophysical characterization of FR50-50 and RAFT50-50 35
   3.2 Microscopy Analysis........36
   3.3 Photostability Study of RAFT50-50 and FR50-50........39
   3.4 Emission vs Absorption 43
   3.5 Photophysical properties of TPEMA monomer, RAFT and FR series 44
4. Photonic and Photovoltaic Characterization 47
   4.1. Radiative Overlap 47
   4.2 Photonic Analysis 50
   4.3 Power Conversion Efficiency, Average Visible-light Transmissivity (AVT) and Light Utilization Efficiency (LUE) of LSC-PV TPEMA-MMA systems 53
Supplementary References........57

1. Characterization of AIE luminogens

1.1 NMR of TPEMA and TPE-COOH

<u>4-(1,2,2-Triphenylvinyl)benzoic acid (TPE-COOH) (**1**)</u>

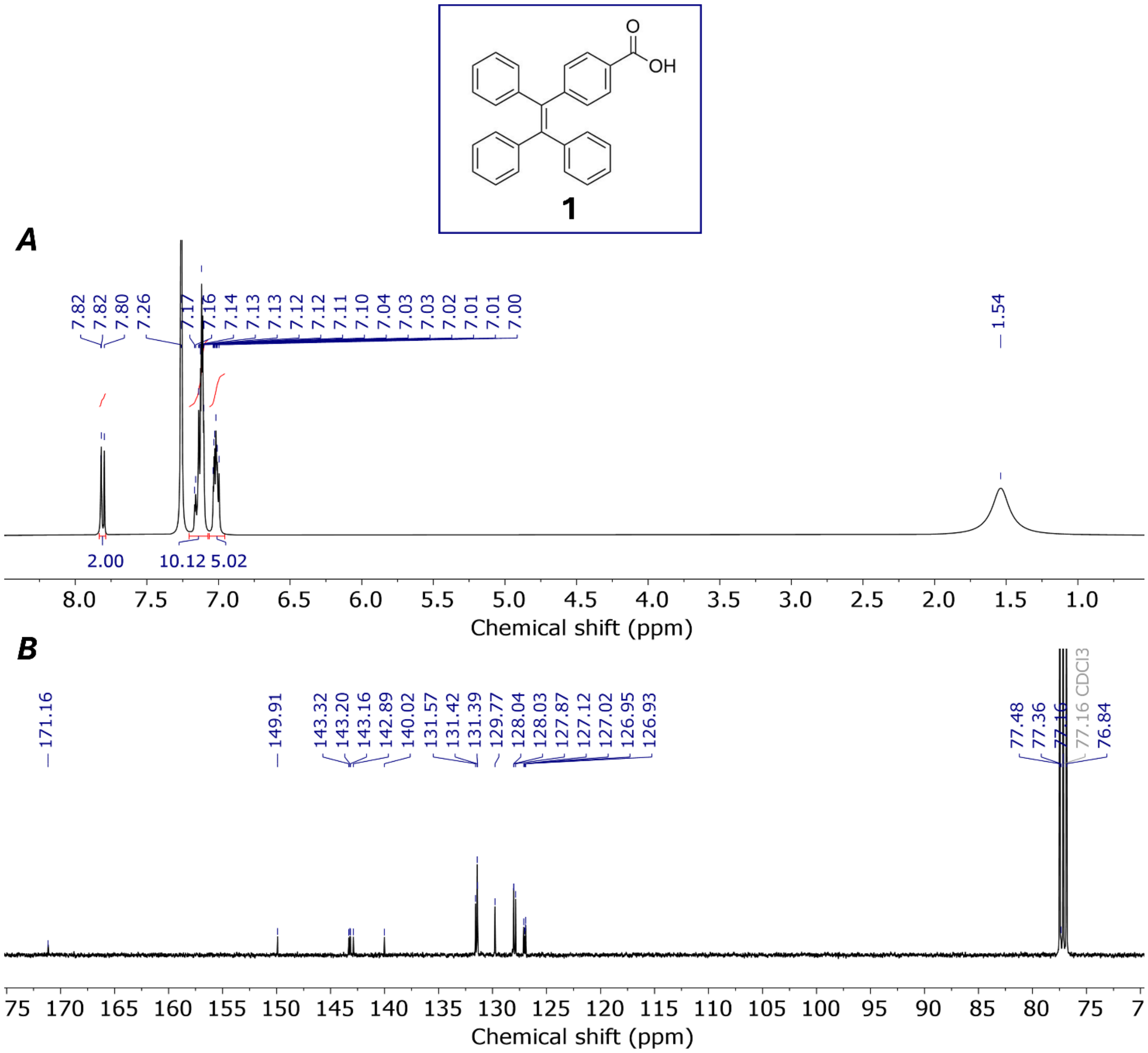

Supplementary Figure 1. ^{1}H-NMR (A) and ^{13}C-NMR (B) in $CDCl_3$ of compound **1**.

2-(methacryloyloxy)ethyl 4-(1,2,2-triphenylvinyl)benzoate) (TPEMA) (**2**)

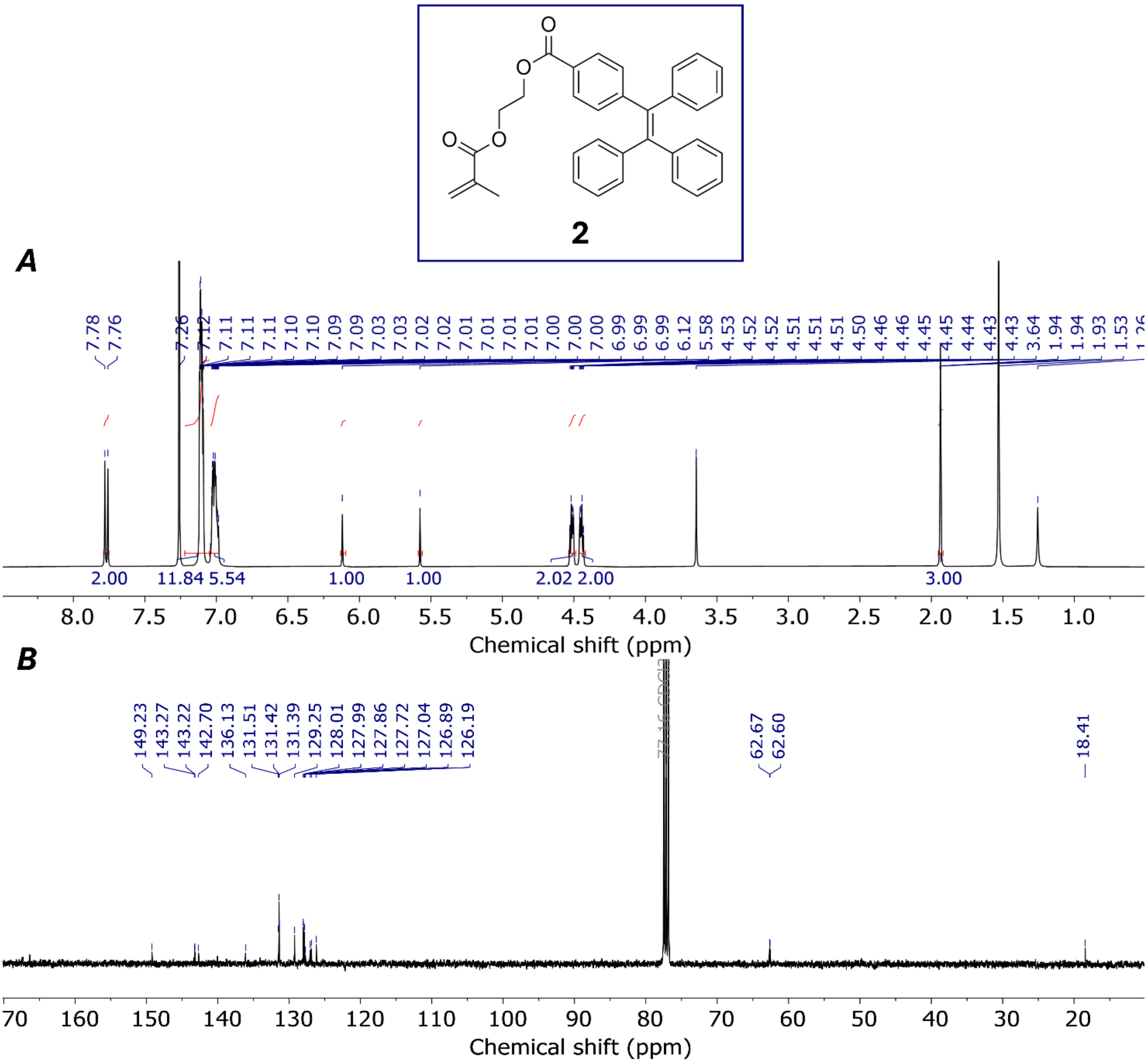

Supplementary Figure 2a. ^{1}H-NMR (A) and ^{13}C-NMR (B) in $CDCl_3$ of compound **2.**

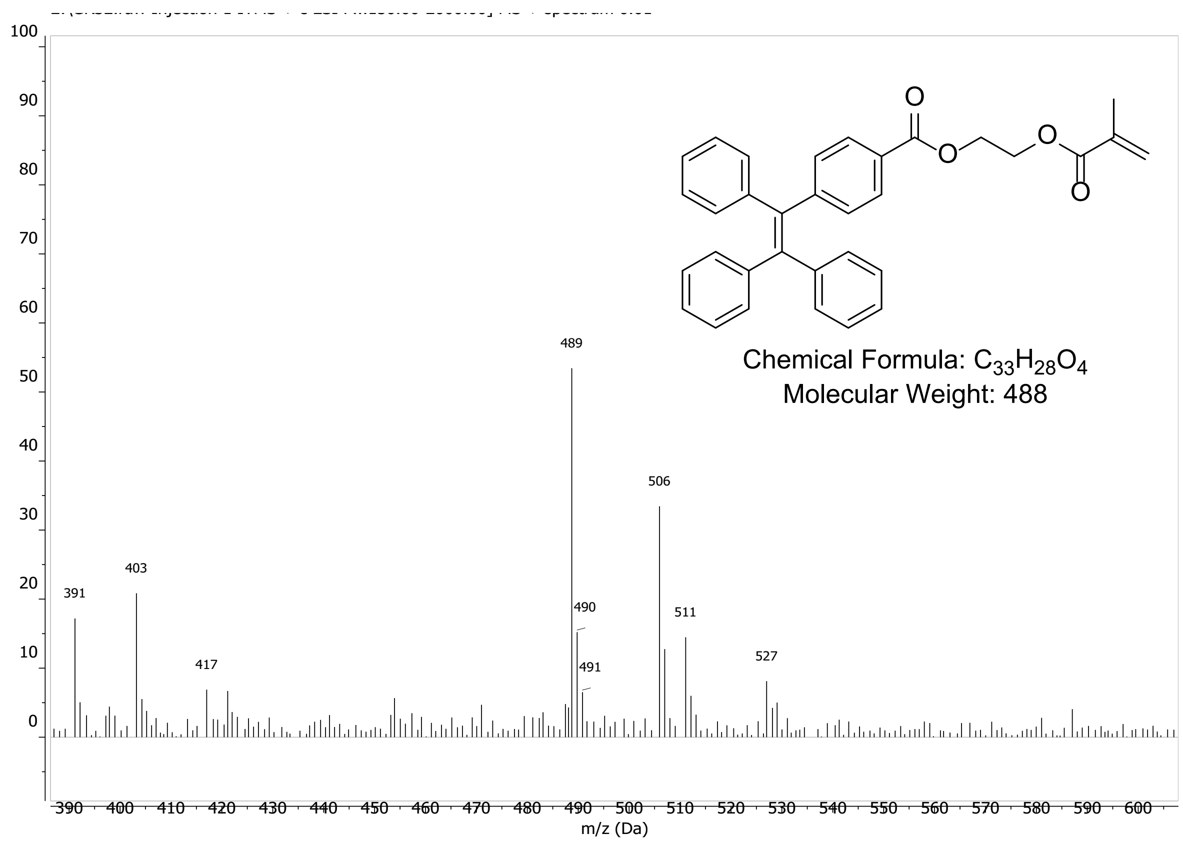

Supplementary Figure 2b. Mass Spectra of compound **2** (ESI positive ions).

1.2 Differential Scanning Calorimetry (DSC) of TPE-COOH and TPEMA

DSC measurements of TPE-COOH and TPEMA are reported in **Supplementary Figure 3**, indicating a melting temperature T_{melt} = 208 °C and T_{melt} = 91 °C, respectively. The TPE molecule exhibits strong intermolecular interactions primarily attributed to π-π stacking, which contributes to its thermal stability and results in a higher melting point. The introduction of a flexible chain when transitioning from TPE-COOH to TPEMA disrupts the π-electron delocalization, thereby diminishing the strength of the intermolecular forces and consequently lowering the T_{melt}. Furthermore, the increase in molecular weight, from 376.46 g/mol for TPE-COOH to 488.58 g/mol for TPEMA, enhances the molecular flexibility, which in turn leads to a reduction in crystallinity and a further decrease in T_{melt}.

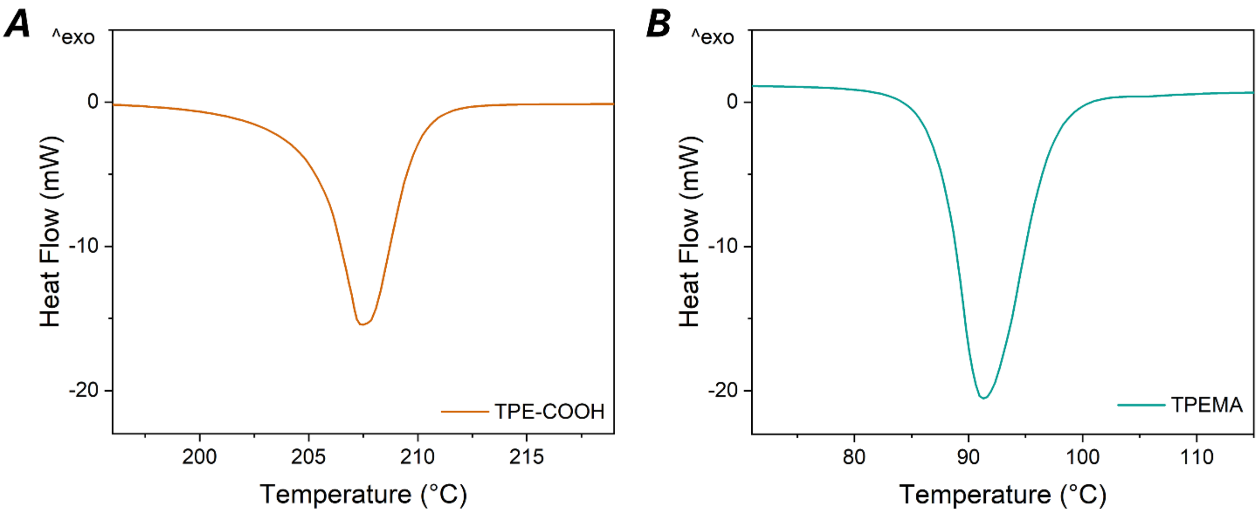

Supplementary Figure 3. DSC traces of TPE-COOH (A) and TPEMA (B).

1.3 Thermogravimetric Analysis (TGA) of TPE-COOH and TPEMA

TGA analyses of TPE-COOH and TPEMA (**Supplementary Figure 4**) revealed an enhanced thermal stability exhibited by TPEMA compared to its precursor TPE-COOH, ascribed to the bulkier structure of the former. Nonetheless, both materials appeared to be stable at least up to 150 °C.

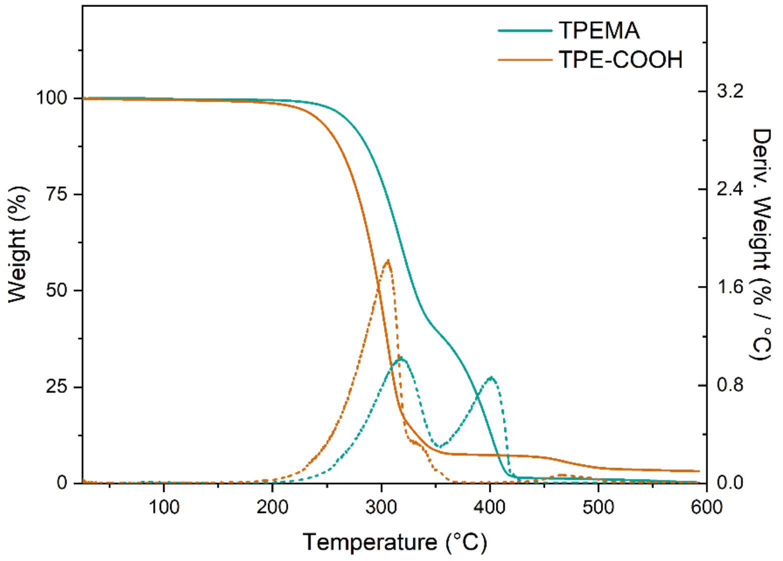

Supplementary Figure 4. TGA analyses of TPEMA and its precursor TPE-COOH.

2 Characterization of polymers

2.1 Synthesis and Characterization of pTPEMA homopolymer by FR and RAFT polymerizations

Synthesis of pTPEMA (FR100) by FR polymerization: a 1 mL vial equipped with a magnetic stirring bar was charged with 4 mg (24 µmol) of AIBN and 200 mg (400 µmol) of TPEMA. Subsequently, the vial was sealed using a rubber septum, followed by the introduction of nitrogen gas. After establishing an inert atmosphere, 880 mL of dry toluene was added. The solution was subjected to bubbling for several minutes to further displace any residual oxygen. Finally, the vial was immersed in an oil bath maintained at a temperature of 90°C, initiating the polymerization. Finally, the resulting copolymer was cooled to room temperature, and the reaction was quenched by oxygen. The final product was obtained by first precipitating it in cold methanol, then filtering it under vacuum, and finally drying it in a vacuum dryer.

Synthesis of pTPEMA (RAFT100) by RAFT polymerization: 100 mg (204µmol) of TPEMA in dry toluene was loaded into a flame-dried, nitrogen purged 4 mL vial and nitrogen bubbling was carried out for about 10 minutes to eliminate the air present in the solution. It was then added under nitrogen to a solution of the AIBN (100µg) and eventually the RAFT agent (571µg) in dry toluene. The reaction vial was positioned on a magnetic stirrer, into an oil bath at 90 °C, allowing the polymerization to proceed. Finally, the resulting copolymer was cooled to room temperature, and the reaction was quenched by oxygen. The final product was obtained by first precipitating it in cold methanol, then filtering it under vacuum, and finally drying it in a vacuum dryer.

2.2 Copolymers characterization

From a preliminary visual inspection conducted under daylight of the FR series, a white appearance was observed. Notably, the FR1-99 copolymer displayed a pronounced crystalline feature, imparting a translucent quality. In contrast, the powders derived from materials containing a higher TPEMA molar content appeared softer, with a greater tendency to stick to substrates. The copolymers synthesized via RAFT polymerization demonstrated analogous behavior, differing only in color. More specifically, the red RAFT agent (CPPA) employed during the syntheses imparted a pink hue to the final material, a feature that was most pronounced in the RAFT1-99 copolymer and gradually transitioned to white as the TPEMA molar concentration increased. Furthermore, when exposed under the UV lamp, the materials resulting from both polymerization processes displayed an emission that red shifted from blue to green with increasing TPEMA molar content (**Supplementary Table 1**).

Supplementary Table 1: Summary table of the FR (left) and RAFT (right) series, including the names of the polymers and their composition, along with the photos under daylight and UV light.

**Name (TPEMA:MMA composition)**	**Under daylight**	**Under UV light**	**Name (TPEMA:MMA composition)**	**Under daylight**	**Under UV light**
**FR1-99 (1:99)**	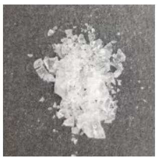	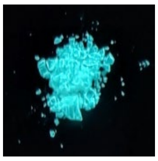	**RAFT1-99 (1:99)**	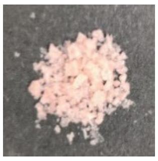	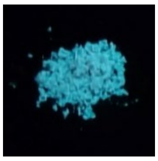
**FR10-90 (10:90)**	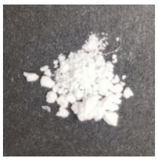	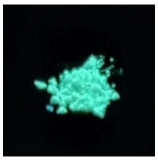	**RAFT10-90 (10:90)**	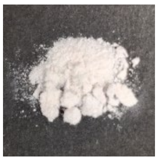	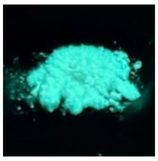
**FR20-80 (20:80)**	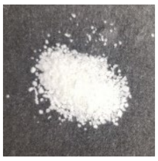	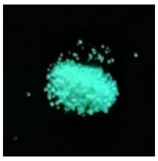	**RAFT20-80 (20:80)**	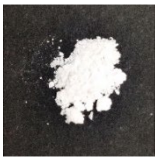	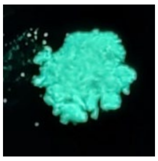
**FR50-50 (50:50)**	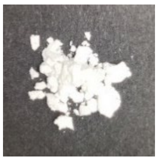	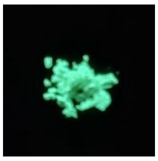	**RAFT50-50 (50:50)**	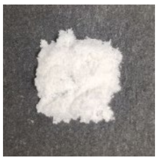	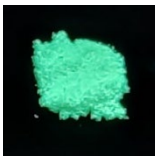
**FR75-25 (75:25)**	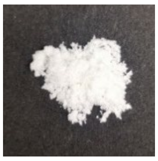	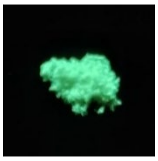	**RAFT75-25 (75:25)**	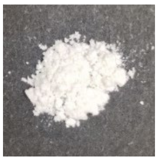	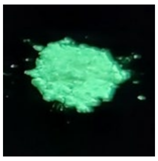

2.3 NMR of RAFT agent, Methyl methacrylate and copolymers

4-Cyano-4-(phenylcarbonothioylthio)pentanoic acid (CPPA) (**3**)

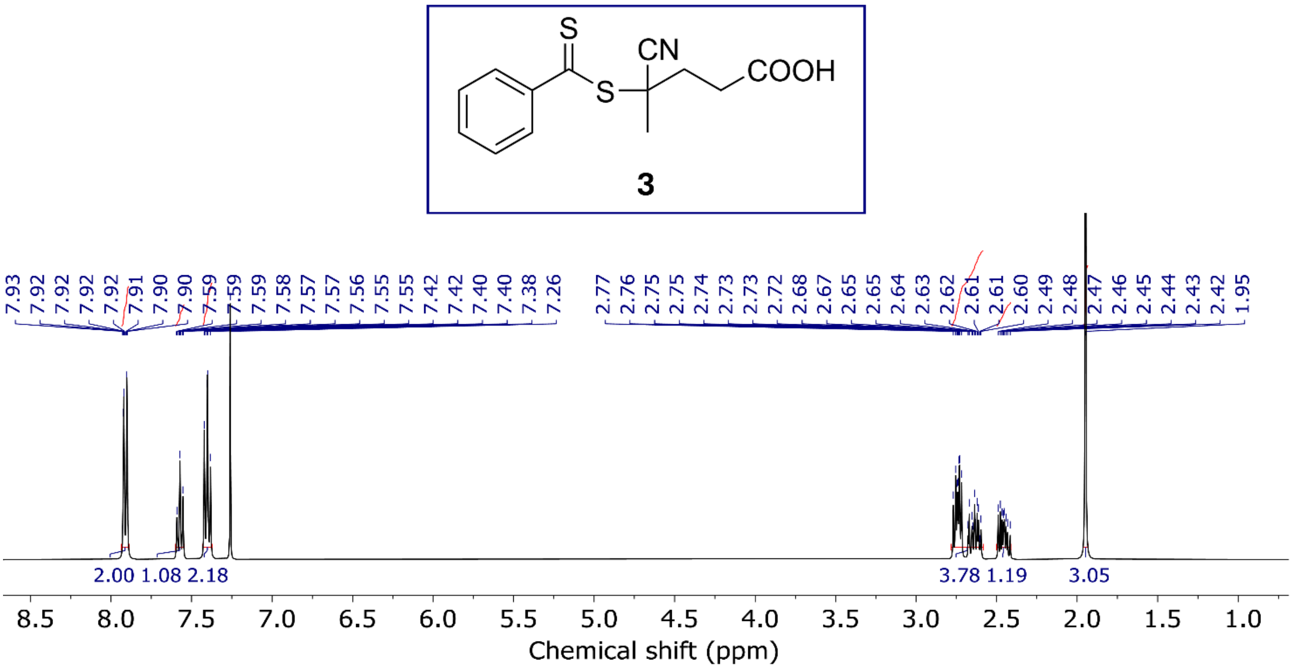

Supplementary Figure 5. ^{1}H-NMR of compound **3.**

Methyl methacrylate (MMA) (**4**)

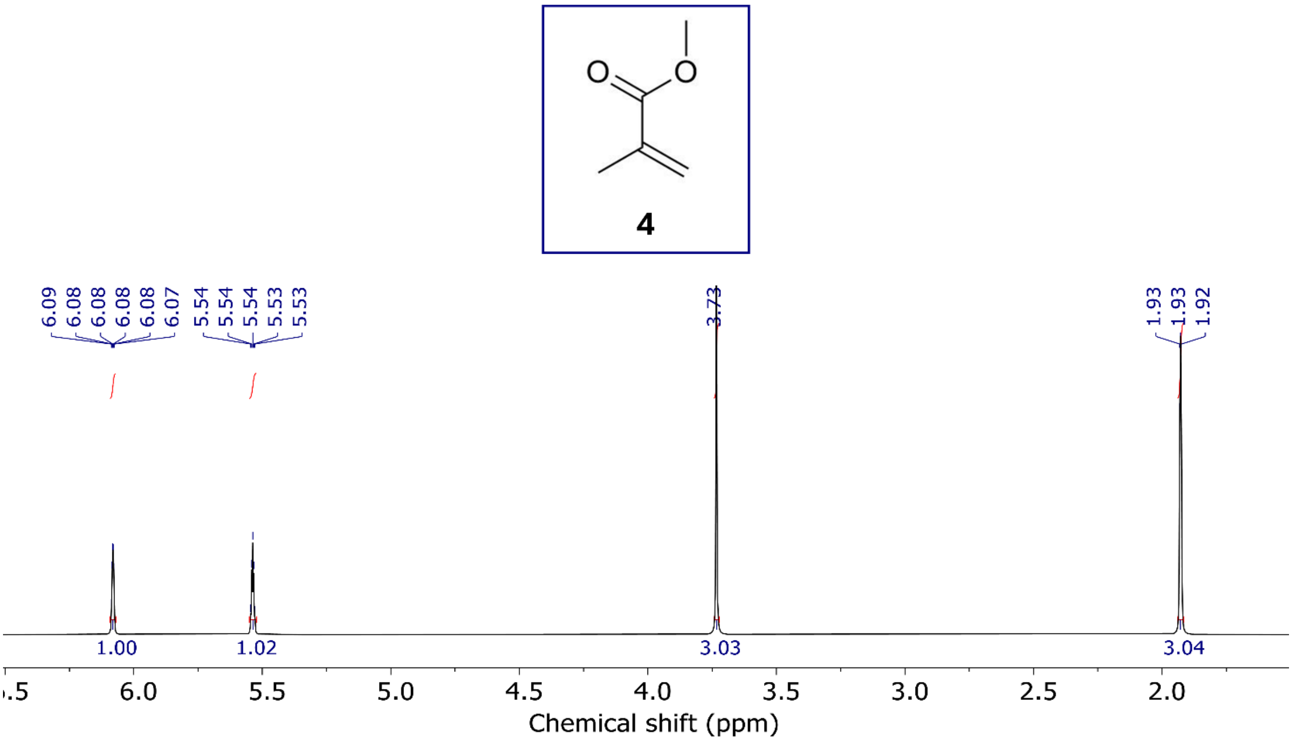

Supplementary Figure 6. ^{1}H-NMR of compound **4**.

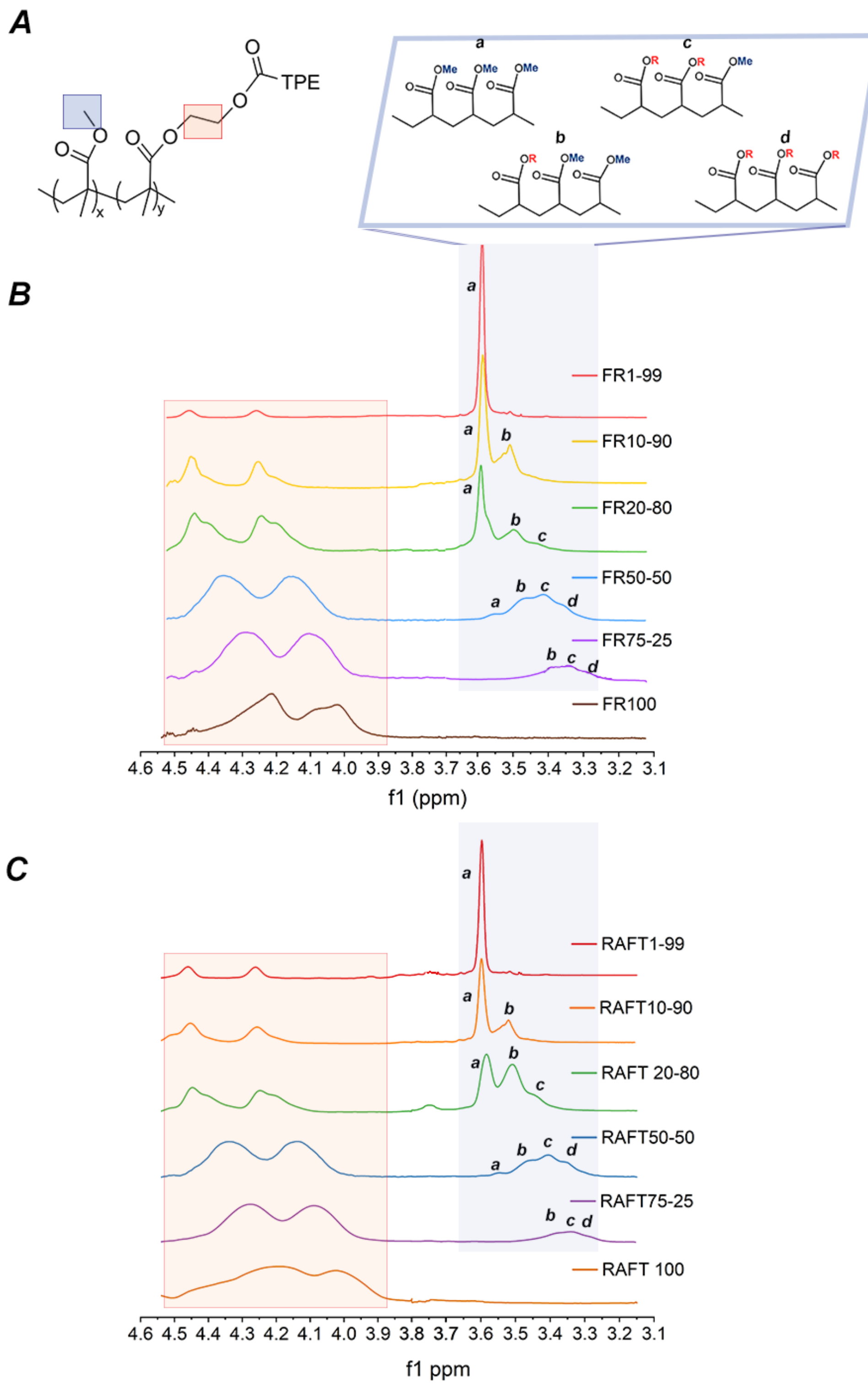

Supplementary Figure 7: Stacked ^{1}H-NMR spectra of the final TPEMA-MMA copolymers and pTPEMA homopolymers obtained via B) FR and C) RAFT polymerization, A) with an

enlargement of the regions including the characteristic peaks of TPEMA ethylene CH_2-CH_2 protons in red and of MMA methoxy O-CH_3, in blue. In blue box the interpretation of the four peaks present in the spectrum between 3.5-3.2 ppm (a,b,c,d) based on the various composition.

The composition of the random copolymers was calculated from the ^{1}H-NMR spectra in terms of molar content of TPEMA, according to **Supplementary Equation 1**:

$$TPEMA\% = \frac{\frac{A_{pTPEMA}}{n.of\ H}}{\frac{A_{pTPEMA}}{n.of\ H} + \frac{A_{pMMA}}{n.of\ H}} \tag{1}$$

where A_{pTPEMA} and A_{pMMA} represent the area-integrals of the NMR signals associated with the methylene protons (CH_2-CH_2) at 4-4.5 ppm and the methoxy protons (O-CH_3) at 3.2-3.5 ppm for PTPEMA and PMMA within the copolymer, respectively. Specifically, the peaks in ^{1}H-NMR spectra were integrated to obtain the value of their underlying area and then normalized by the number of hydrogen atoms associated with each signal ($n.of\ H$).

Supplementary Table 2. Progression of FR and RAFT polymerizations and final composition.

Copolymer	Time (h)	Conversion (%) MMA/TPEMA [1]	Composition TPEMA (%) [2]
**FR1-99**	23	99/99	1
**FR10-90**	95	99/97	11
**FR20-80**	76	97/96	18
**FR50-50**	53	96/94	50
**FR75-25**	72	94/87	72
**FR100**	79	0/96	100
**RAFT1-99**	22	99/99	1
**RAFT10-90**	72	99/86	12
**RAFT20-80**	72	98/83	24
**RAFT50-50**	72	99/84	51
**RAFT75-25**	121	99/86	71
**RAFT**	72	0/NA	100

[1] The conversion of MMA and TPEMA was calculated via Supplementary Equation 2 and 3, respectively. [2] The composition was calculated with Supplementary Equation 1.

NMR Characterization of RAFT and FR Copolymer Microstructure

To gain further insights into the microstructure and monomer distribution within the RAFT copolymers, we performed detailed NMR analysis on two representative samples: RAFT50–50 and RAFT20–80. The full NMR datasets include ^{1}H, ^{13}C, and two-dimensional HSQC spectra. Stacked ^{13}C NMR spectra for the two samples are shown in **Supplementary Figure 8** in the regions of 46–43 ppm (assigned to methylene carbons in α-position to the ester groups) and 181–164 ppm (assigned to the carbonyl carbons of the methacrylate backbone). These regions were selected as they are expected to be most sensitive to the local monomer sequence environment and thus potentially informative regarding the copolymer architecture.

The spectra reveal some differences in chemical shift patterns and signal intensity consistent with the varying TPEMA content. However, due to significant peak overlap and the inherently low signal-to-noise ratio of ^{13}C NMR spectra for polymers with chemically similar repeating units, it is not possible to make definitive assignments of triad sequences or quantify the degree of randomness versus blockiness. This limitation is especially pronounced given the structural similarity between MMA and TPEMA, which causes their respective carbon environments to resonate in nearly identical spectral regions.

The accompanying HSQC spectra (**Supplementary Figures** 11, 14, 17, 20) confirm the presence of both monomer types and support the structural assignments observed in the one-dimensional spectra. While the NMR data do not allow quantitative analysis of sequence distribution, they support the successful incorporation of both comonomers in proportions consistent with the feed ratios and ^{1}H NMR integrations.

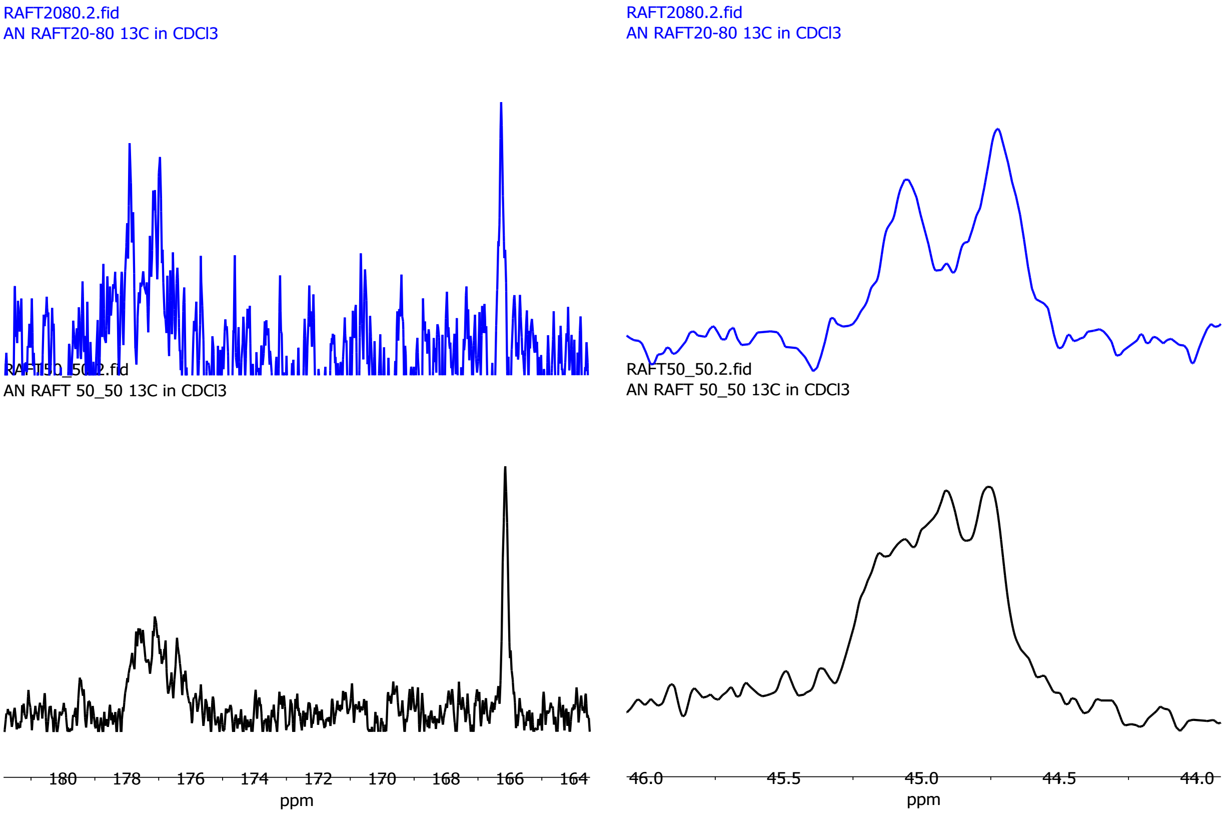

Supplementary Figure 8: Stacked ^{13}C NMR spectra (in $CDCl_3$) of copolymers RAFT20-80 (blue line) and RAFT50-50 (black line) in the region of 180-164 ppm (left, signals corresponding to the -CO- moieties) and 46-44 ppm (right, signals corresponding to the -CH- groups in α- to the carboxylic esters).[1] Although the ^{13}C NMR spectra do not allow for an unambiguous assignment of the microstructural sequence due to significant signal overlap and low intensity, qualitative differences can still be observed. In particular, changes in the shape and intensity of the single component of the signals suggest variations in the microstructural arrangement of the copolymers, supporting the hypothesis of altered monomer distribution along the polymer backbone.

RAFT20-80

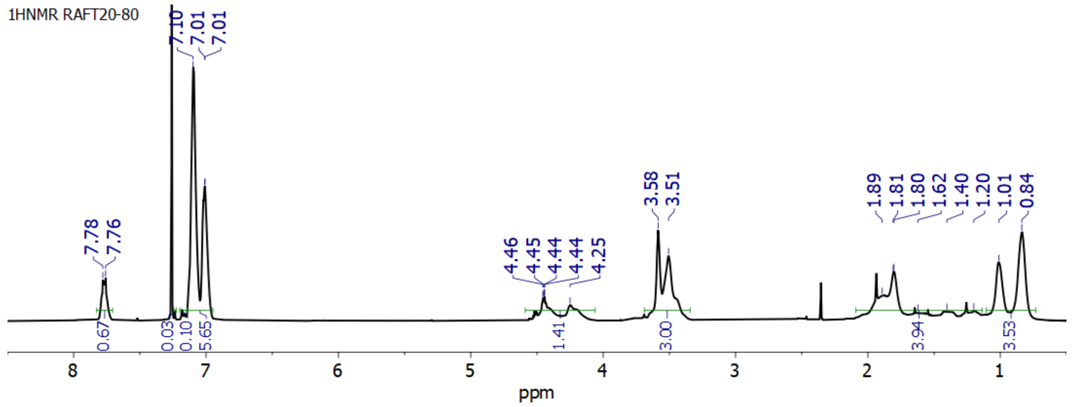

Supplementary Figure 9: ^{1}H NMR (400 MHz, $CDCl_3$) of copolymer RAFT20-80

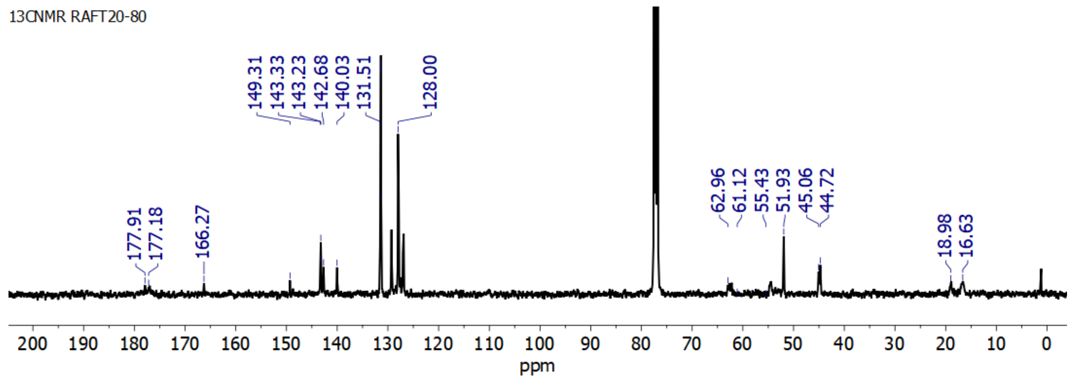

Supplementary Figure 10: ^{13}C NMR (100 MHz, $CDCl_3$) of copolymer RAFT20-80

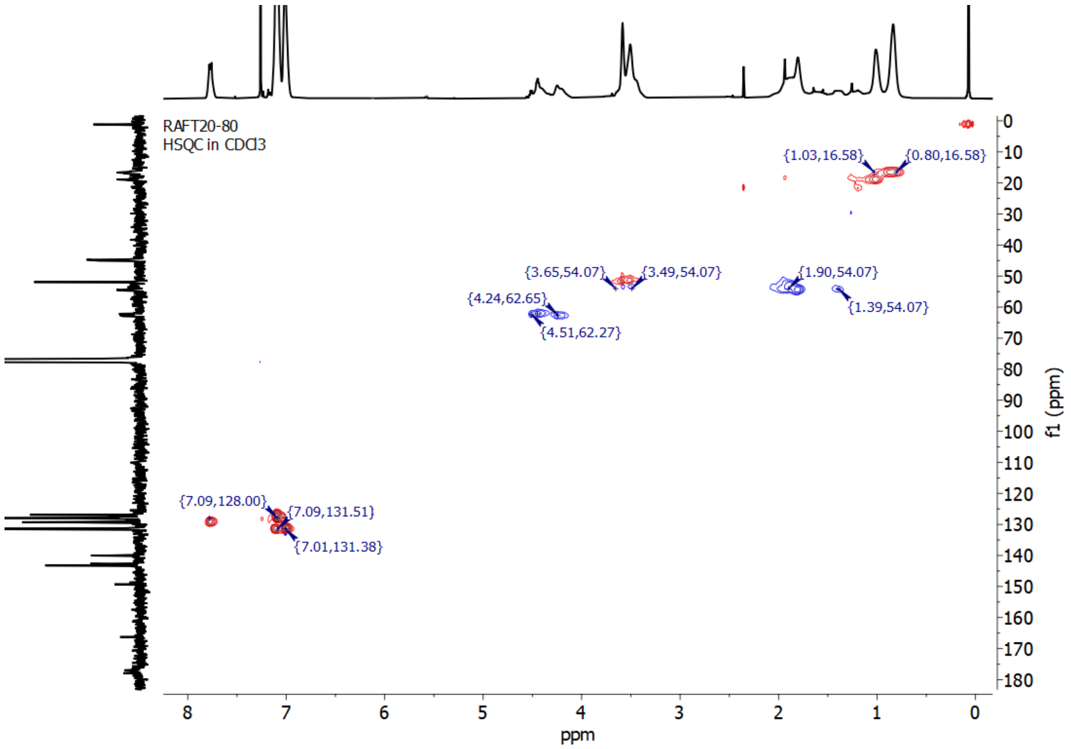

Supplementary Figure 11: HSQC of copolymer RAFT20-80

RAFT50-50

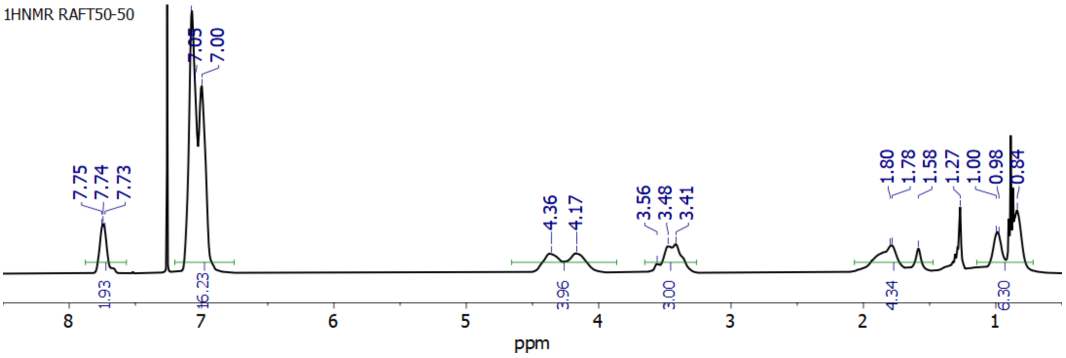

Supplementary Figure 12: ^{1}H NMR (400 MHz, $CDCl_3$) of copolymer RAFT50-50

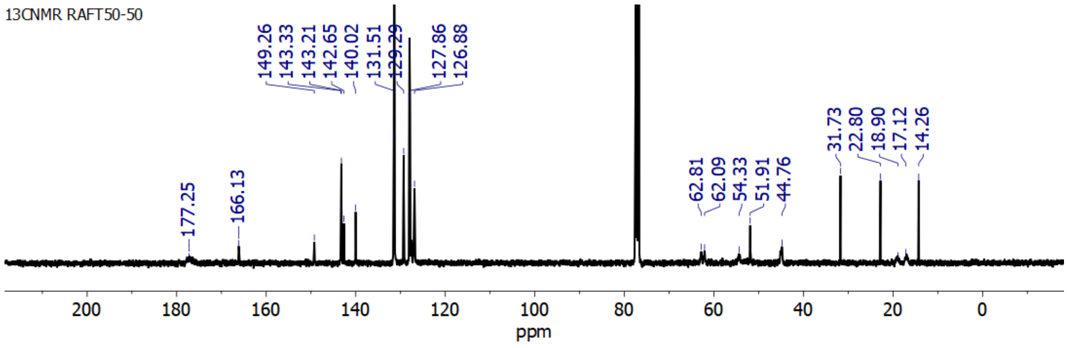

Supplementary Figure 13: ^{13}C NMR (100 MHz, $CDCl_3$) of copolymer RAFT50-50

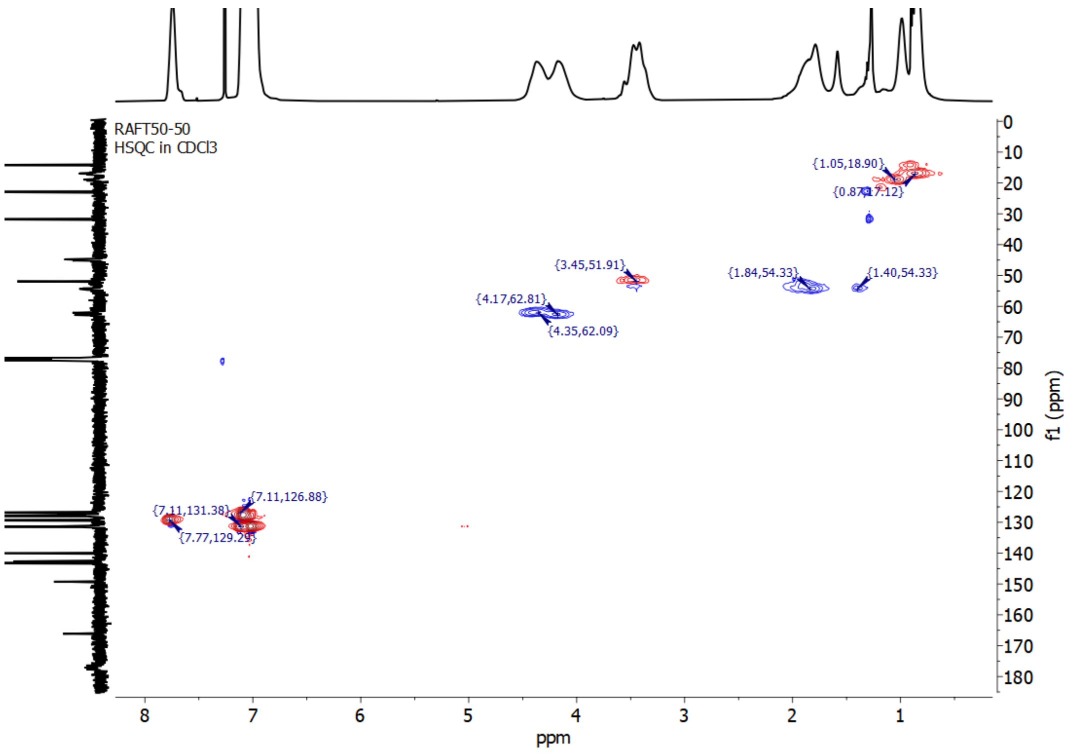

Supplementary Figure 14: HSQC of copolymer RAFT50-50

FR20-80

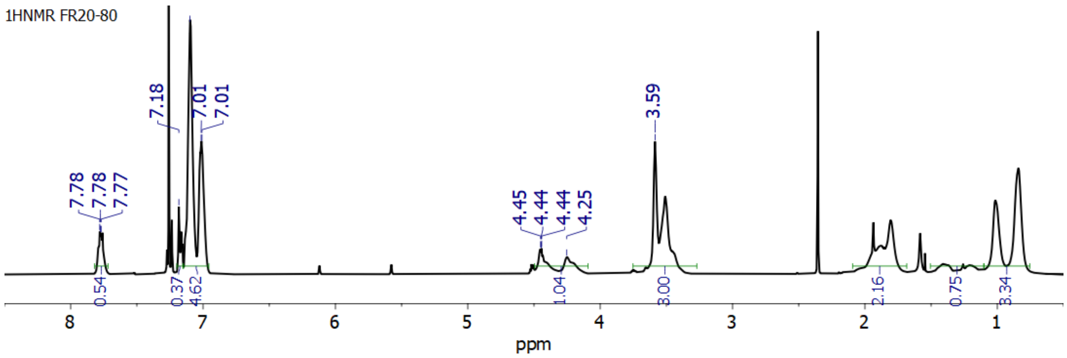

Supplementary Figure 15: ^{1}H NMR (400 MHz, $CDCl_3$) of copolymer FR20-80

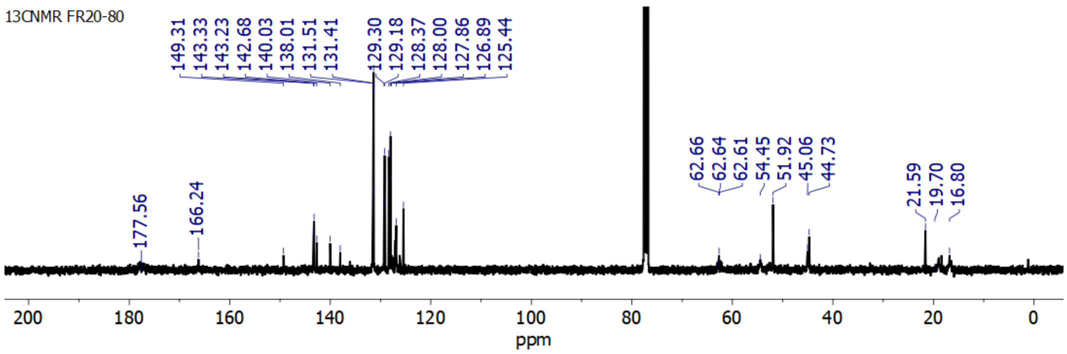

Supplementary Figure 16: ^{13}C NMR (100 MHz, $CDCl_3$) of copolymer FR20-80

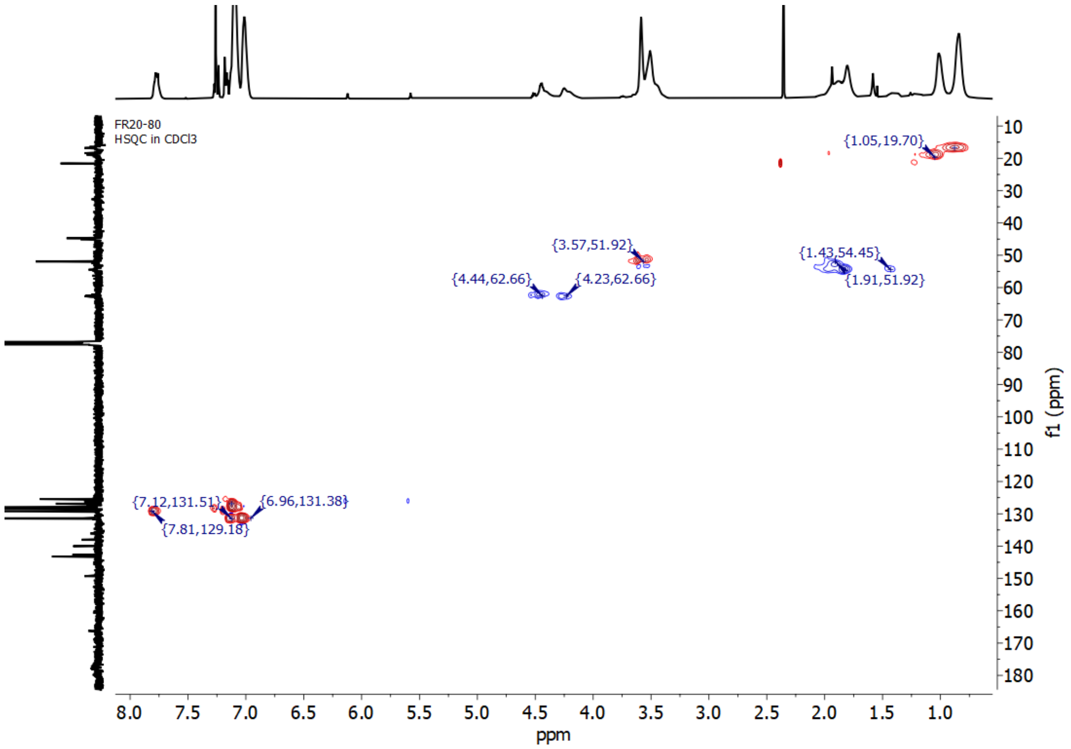

Supplementary Figure 17: HSQC of copolymer FR20-80

FR50-50

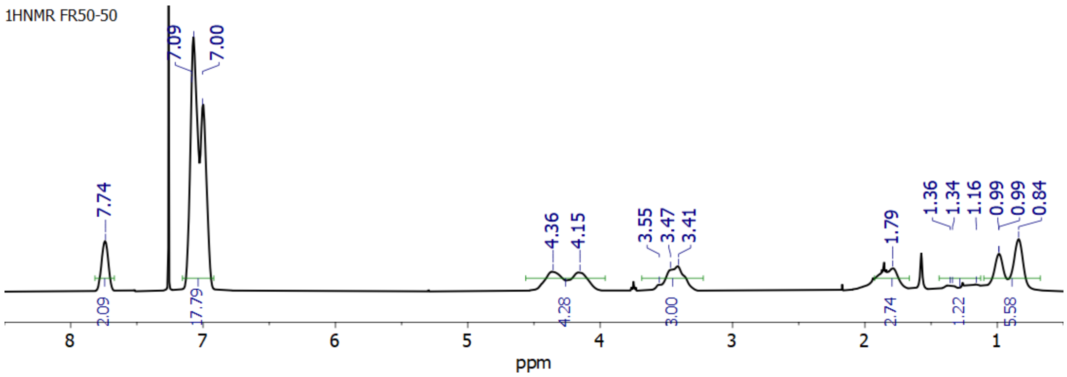

Supplementary Figure 18: ^{1}H NMR (400 MHz, $CDCl_3$) of copolymer FR50-50

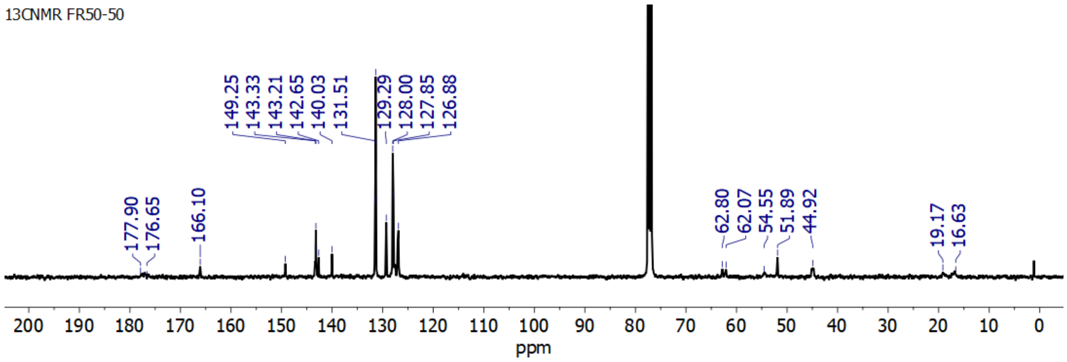

Supplementary Figure 19: ^{13}C NMR (100 MHz, $CDCl_3$) of copolymer FR50-50

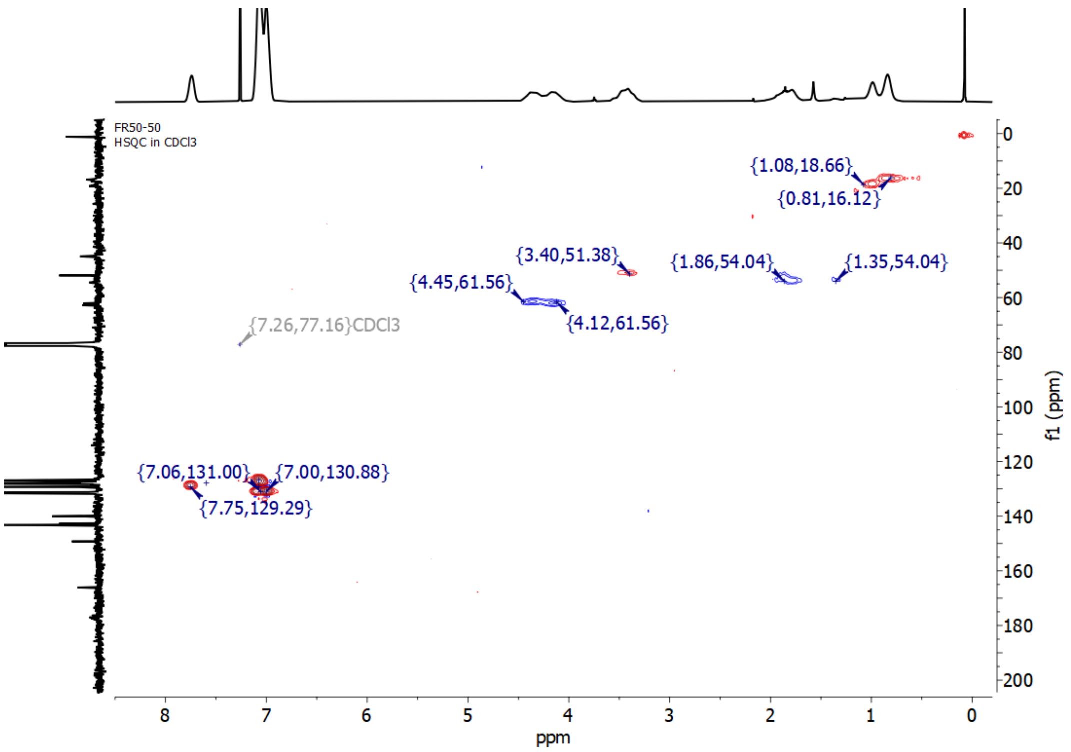

Supplementary Figure 20: HSQC of copolymer FR50-50

2.4 Reactivity Study

A series of TPEMA-MMA copolymers with composition equal to 10-90, 15-85, 20-80, 25-75 and 50-50, was synthesized via FRP to conduct a reactivity study on the monomers composing the copolymers proposed in this work. The investigation was based on the theoretical approach proposed by Mayo-Lewis. In order to perform the study at low yield values (<10%) where the Mayo-Lewis equation holds true, samples of 10 µl were taken from the reaction vessel at regular intervals of time equal to 5 minutes to check the conversion of the monomers by means of ^{1}H-NMR analyses. At very low times from the start of the reaction, the copolymers in formation exhibited not clearly detectable signals in the ^{1}H-NMR spectrum, whereas at high times the conversions of the monomers approached already too high values. Therefore, on the basis of the monomer conversions to be kept low (<10%) and the reliability of the integrals, a 10 minutes reaction time was selected as the most suitable for subsequent computations.

The conversions of the monomers were calculated through the following **Supplementary Equation 2** and **3**:

$$conversion\ of\ MMA\ (\%) = \frac{\frac{A_{MMA,cop}}{n.\ of\ H}}{\frac{A_{MMA,cop}}{n.\ of\ H} + \frac{A_{MMA}}{n.\ of\ H}} \cdot 100 \tag{2}$$

$$conversion\ of\ TPEMA\ (\%) = \frac{\frac{A_{TPEMA,cop}}{n.\ of\ H}}{\frac{A_{TPEMA,cop}}{n.\ of\ H} + \frac{A_{TPEMA}}{n.\ of\ H}} \cdot 100 \tag{3}$$

where $A_{MMA,cop}$ and $A_{TPEMA,cop}$ represent the areas related to the signals of MMA and TPEMA inside the copolymer, respectively, while A_{MMA} and A_{TPEMA} represent the areas related to MMA and TPEMA monomers. Each area is divided by the number of hydrogen atoms relative to the signal ($n.\ of\ H$).

The molar fraction of TPEMA monomer in the input mixture (f_1) and in the output copolymer (F_1) were retrieved from the ^{1}H-NMR spectrum. Subsequently, the composition of the inlet monomer mixture μ and the instantaneous composition of the output copolymer Π were recovered through the following equations (**Supplementary Equation 4** and **5**):

$$\mu = \frac{f_1}{f_2} \tag{4}$$

$$\Pi = \frac{F_1}{F_2} \tag{5}$$

Finally, the linearization of the Mayo-Lewis equation according to the Fineman-Ross method is expressed as follows (**Supplementary Equation 6**):

$$G = Hr_1 - r_2 \tag{6}$$

where $H = \left(\frac{\mu^2}{\Pi}\right)$ and $G = \left(\frac{\Pi - 1}{\Pi}\right)\mu$; the reactivity ratio of TPEMA (r_1) corresponds to the slope of the fitting line, whereas the reactivity ratio of MMA (r_2) corresponds to the intercept with the y-axis. The values extrapolated from experimental data are reported in **Supplementary Table 3**.

In the specific case of Kelen-Tüdos refined the Fineman-Ross equation by the introduction of an arbitrary constant (α) (**Supplementary Equation 7**) to distribute the data more uniformly

and eliminate bias from certain experimental data points.[3–5] The previously defined values, G and H can be modified, giving values ξ and η (**Supplementary Equation 8-9**) where r1 and r2 are the reactivity ratios of monomers 1 and 2 and G and H are variables defined from the Fineman Ross technique. H_{min} and H_{max} are the minimum and maximum values of H determined from the data.

$$\alpha = H_{min} * H_{max} \tag{7}$$

$$\xi = \frac{H}{\alpha + H} \tag{8}$$

$$\eta = \frac{G}{\alpha + H} \tag{9}$$

Finally, the linearization of the Mayo-Lewis equation according to the Kelen Tüdos method is expressed as follows (**Supplementary Equation 10**):

$$\eta = \left(r_1 + \frac{r_2}{\alpha}\right)\xi - \frac{r_2}{\alpha} \tag{10}$$

Reactivity ratios r1 and r2 can be calculated by plotting ξ versus η for each experiment. This yields a straight line, where $\eta = r_1$ at $\xi = 1$ and $\eta = -r_2 / \alpha$ at $\xi = 0$. The reactivity ratio of TPEMA (r_1) can be calculated from the slope of the fitting line, whereas the reactivity ratio of MMA (r_2) can be calculated from the intercept with the y-axis. The values extrapolated from experimental data are reported in **Supplementary Table 3**.

Supplementary Table 3: Monomer conversions and Mayo-Lewis parameters extrapolated from experimental data (at yield values < 10%).

TPEMA-MMA	Conv. TPEMA (%)	Conv. MMA (%)	f_{TPEMA}	F_{TPEMA}	f_{MMA}	F_{MMA}	μ	Π	Fineman Ross		Kelen Tüdos	
									H	G	ξ	η
**10-90**	5	4	0.116	0.158	0.884	0.842	0.131	0.187	0.092	-0.568	0.265	-1.642
**15-85**	9	7	0.185	0.223	0.815	0.777	0.227	0.287	0.179	-0.563	0.413	-1.300
**20-80**	9	8	0.220	0.258	0.780	0.742	0.282	0.348	0.228	-0.528	0.472	-1.094
**25-75**	10	9	0.287	0.318	0.713	0.682	0.403	0.465	0.349	-0.463	0.578	-0.767
**50-50**	9	10	0.580	0.544	0.420	0.456	1.382	1.192	1.601	0.223	0.863	0.120

2.5 Gel Permeation Chromatography (GPC) of copolymers

GPC analyses were performed to determine the average molecular weights of the copolymers and their polydispersity index (*Đ*). The GPC results related to TPEMA-MMA random copolymers synthesized via FRP and RAFT polymerization are reported in **Supplementary Table 4**. As expected, the final molecular weights of the FR series were generally higher compared to the RAFT series. In addition, RAFT polymerization permitted a major control over the *Đ*, with values falling in the range of 1.15-1.21. Nonetheless, good results were obtained via FRP as well, with PDI ranging from 1.66 to 1.94.

Supplementary Table 4. GPC results of FR and RAFT series.

**Copolymers**	M_n **(Kg/mol)**	M_w **(Kg/mol)**	M_p **(Kg/mol)**	***Đ***
**FR1-99**	27.7	50.3	47.2	1.82
**FR10-90**	18.3	34.9	33.3	1.91
**FR20-80**	11.5	19.0	17.9	1.66
**FR50-50**	8.5	16.4	16.6	1.94
**FR75-25**	11.5	20.2	17.3	1.76
**RAFT1-99**	11.1	12.8	14.3	1.15
**RAFT10-90**	8.7	10.6	12.0	1.21
**RAFT20-80**	11.8	14.3	16.2	1.21
**RAFT50-50**	15.5	18.3	19.9	1.18
**RAFT75-25**	14.6	17.6	19.1	1.21

2.6 Differential Scanning Calorimetry (DSC) of copolymers

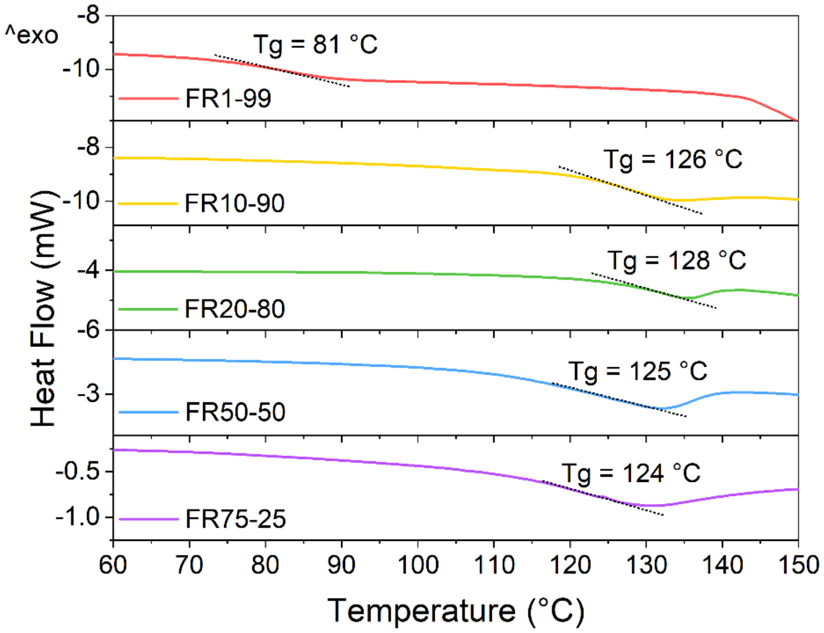

Supplementary Figure 21. DSC traces of TPEMA-MMA copolymers via FRP.

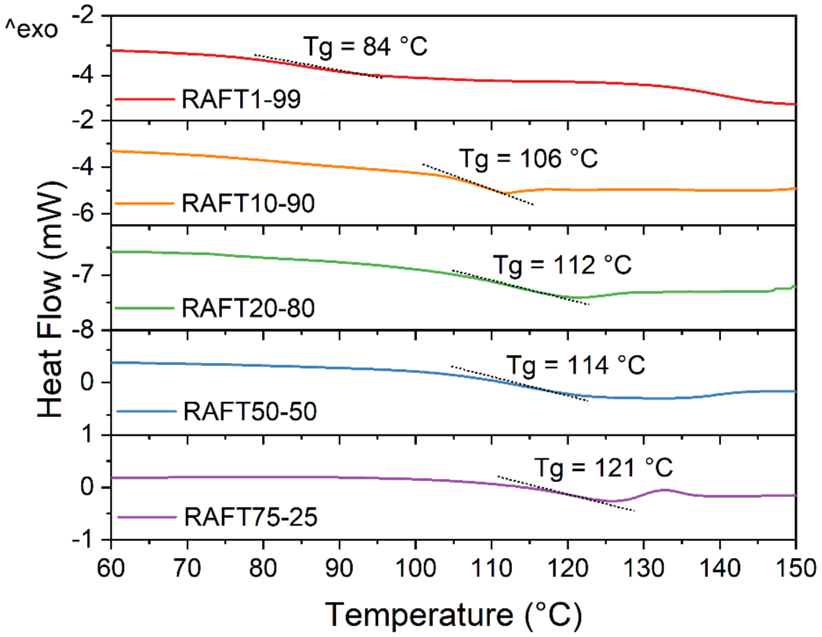

a

Supplementary Figure 22. DSC traces of TPEMA-MMA copolymers via RAFT polymerization.

The glass transition temperature (T_g) exhibited an increasing trend as the TPEMA molar concentration increased, across both synthesis methodologies (**Supplementary Figures 21**and **22**). The results are consistent since TPEMA displays a bulkier structure compared to MMA, resulting in enhanced rigidity and a more pronounced glassy feature. Specifically, in the case of the materials synthesized via FR, Tg increased until reaching a sort of plateau, with all the Tg converging around the same value. Conversely, in the second case, the second synthesis method demonstrated a more gradual and uniform increase in T_g. This finding highlights the significant impact of RAFT polymerization in controlling not only the molecular weight, but also the macromolecular architecture and the associated properties of the copolymers.

Furthermore, for random copolymers, the Tg can be also theoretically calculated using the Fox–Flory (**Supplementary Equation 11**)[6]:

$$\frac{1}{T_g} = \frac{w_1}{T_{g,1}} + \frac{w_2}{T_{g,2}} \tag{11}$$

where w_1 and w_2 are weight fractions of components 1 and 2, respectively, inside the copolymer; $T_{g,1}$ and $T_{g,2}$, instead, represent the glass transition temperatures of the corresponding distinct homopolymers. In this context, the value of Tg corresponding to poly-TPEMA was experimentally determined through DSC analysis of the TPEMA homopolymer synthesized via free radical polymerization, and resulted equal to 123 °C. Specifically, a TPEMA homopolymer was synthesized via free radical polymerization according to the same experimental conditions and setup already described for the TPEMA-MMA copolymers (**Section 2.2, main text**). Conversely, the T_g of poly-MMA was retrieved from the range of values provided by scientific literature, and an average T_g of 100 °C was selected. In **Table 1**

(**main text**), the values of Tg experimentally obtained through DSC analyses and theoretically retrieved ($T_{g,theo}$) using **Supplementary Equation 10** are reported. As can be noted, the T_g corresponding to the RAFT series are in line with the predicted values from theory, as opposed to those related to the FR series, further demonstrating the potentiality of such type of controlled radical polymerization process.

2.7 Thermogravimetric Analysis (TGA) of copolymers

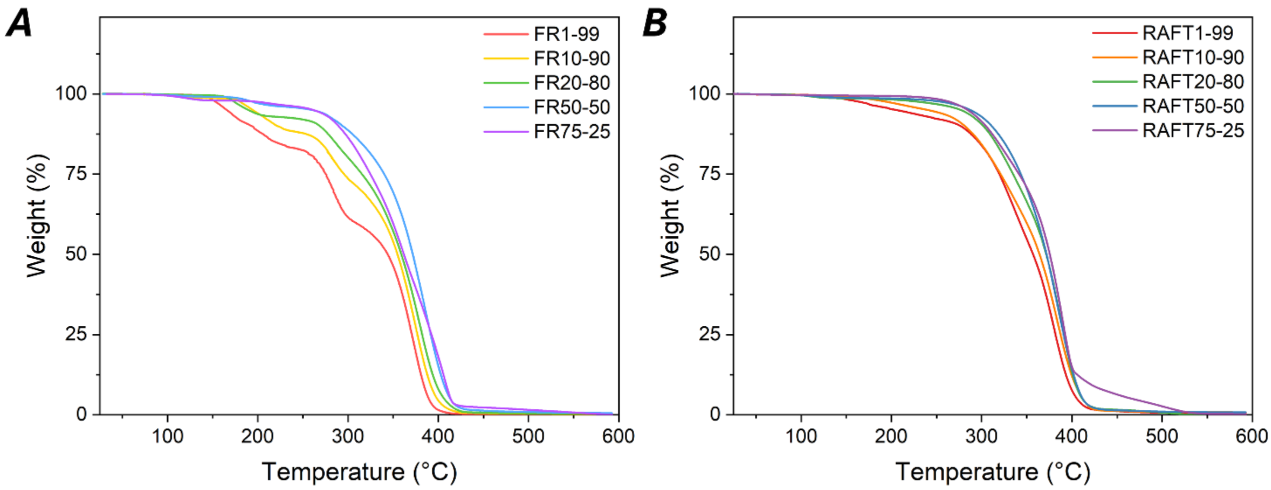

Supplementary Figure 23. TGA analyses of TPEMA-MMA copolymers via FR (A) and RAFT approaches (B).

In both FRP and RAFT polymerization processes, the copolymers resulted to be stable at least up to 100 °C, rendering them viable for LSC device application. Furthermore, as expected, the thermal stability became more pronounced with increasing TPEMA molar content inside the copolymers, owing to its bulky structure (**Supplementary Figure 23**). Moreover, a controlled synthesis as the RAFT polymerization allows for a higher thermal stability, compared to FRP (**Supplementary Figure 24**).

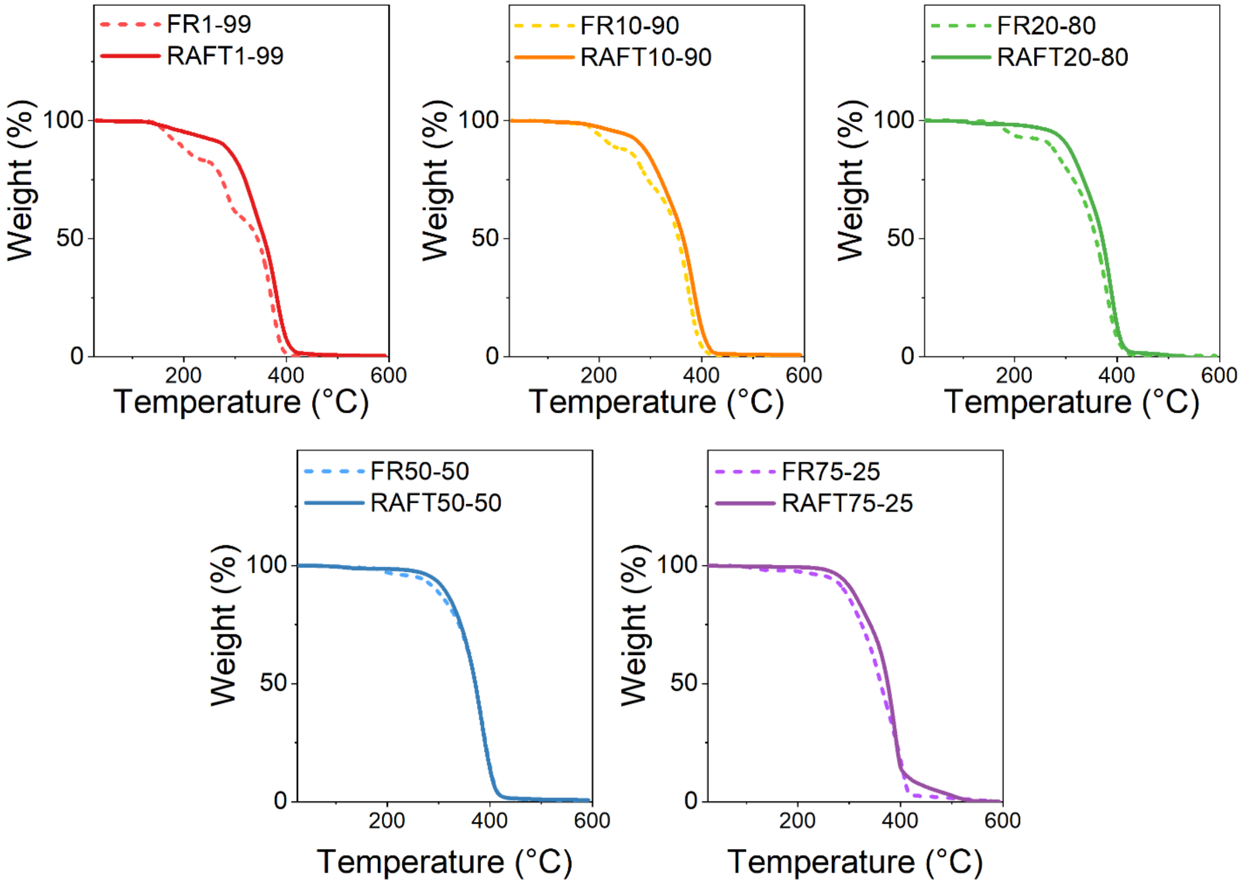

Supplementary Figure 24. Comparison between TGA analyses of FRP and RAFT polymerization.

2.8 Profilometer Analysis

The thickness of the films (**Supplementary Table 5**) was measured using a stylus profilometer. The values related to the RAFT series exhibited minimal variation, falling within the range of 0.7-0.8 μm. Therefore, in subsequent analyses such as absorption measurements, the impact of thickness on the results was considered negligible. Conversely, the thicknesses obtained for the FR series displayed a wider range of values, spanning from 0.62 to 1 μm, hence further considerations were done in absorption measurements. Specifically, the absorbance was normalized by the thickness, being this a parameter appearing in the Lambert Beer law (see **Section 2.3, main text**).

Supplementary Table 5. Film thicknesses of TPEMA-MMA random copolymers.

Copolymer	Thickness (μm)	Copolymer	Thickness (μm)
**FR1-99**	0.910 ± 0.006	**RAFT1-99**	0.808 ± 0.010
**FR10-90**	0.953 ± 0.020	**RAFT10-90**	0.704 ± 0.009
**FR20-80**	1.047 ± 0.018	**RAFT20-80**	0.724 ± 0.024
**FR50-50**	0.620 ± 0.017	**RAFT50-50**	0.767 ± 0.017
**FR75-25**	0.703 ± 0.018	**RAFT75-25**	0.766 ± 0.014

3. Photophysical Characterization

3.1 Photophysical characterization of FR50-50 and RAFT50-50

To explore the AIE characteristics of polymers, their photophysical properties were analyzed in mixtures of THF-H_2O with different water contents. UV-VIS and PL emission spectra were recorded for selected polymers from the FR and RAFT series with a 50% molar content of TPEMA (FR50-50 and RAFT50-50), demonstrating their AIE behavior. Specifically, solutions with water volume fractions (f_w) of 0%, 35%, and 95% were tested, as illustrated in **Supplementary Figure 11**. In this solvent system, THF serves as an effective solvent for the copolymers, whereas water acts as a poor solvent, triggering aggregation at higher water fractions. This setup allows for a qualitative assessment of the AIE effect, where the emission intensity is expected to increase with rising water content, facilitating molecular aggregation, as shown in **Supplementary Figure 25A**. As depicted in **Supplementary Figures 25B** and **25C**, the copolymers at 0% and 35% f_w do not exhibit emissivity, whereas at 95% f_w, the emission intensity increases by 300 times. Additionally, Panel B presents the fluorescence of 95% fw and the cast film, normalized for comparison.

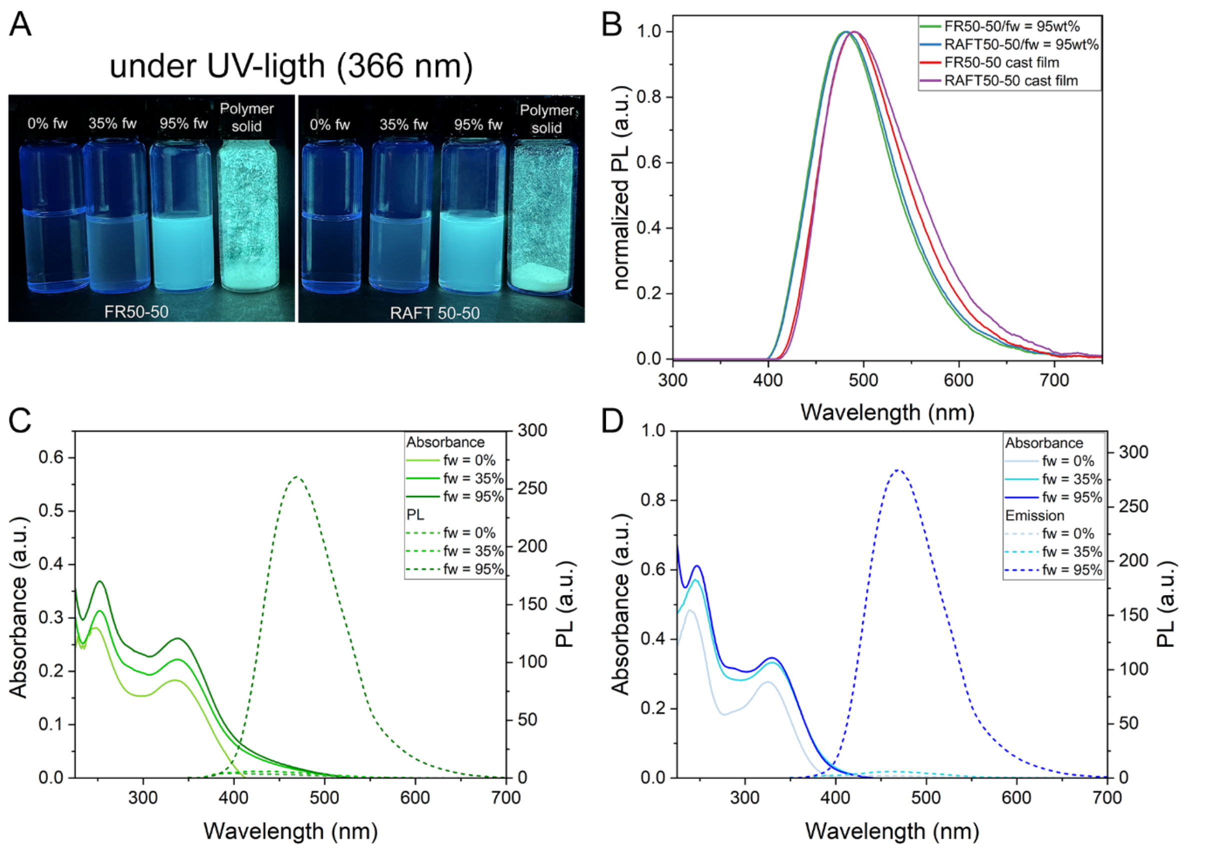

Supplementary Figure 25. A) Photographic images of the solutions of FR 50-50 and RAFT 50-50 and the vial with the polymer (powder form) under a TLC UV lamp (@ 366 nm), B) normalized emission of f_w 95% and cast film of both FR 50-50 and RAFT 50-50, C) UV-VIS and photoluminescence emission behavior of C) FR 50-50 and D) RAFT 50-50 in THF-H_2O mixtures with different volume fractions of water (f_w = 0%, 35%, 95%).

3.2 Microscopy Analysis

To investigate potential microphase separation, aggregation of TPEMA units, or structural heterogeneities between the copolymer films prepared *via* FR polymerization and RAFT polymerization, we performed both bright-field and fluorescence microscopy on FR50-50 and RAFT50-50 thin films as it is visible in **Supplementary Figure 26**. Images were acquired at

two magnifications (10× and 40×), under both bright-field and UV excitation (360 nm) modes. Bright-field microscopy was used to detect possible phase separation or large-scale heterogeneities in the polymer matrix, while fluorescence microscopy was employed to visualize the spatial distribution of the TPEMA units, whose aggregation would lead to local enhancement or variation in emission intensity due to their AIE properties.

Across all conditions and imaging scales, both FR and RAFT samples displayed smooth film morphology, without any visible phase-separated domains. In fluorescence images, the emission was evenly distributed over the entire field of view, with no detectable intensity gradients, bright spots, or domain-like structures that would suggest preferential clustering or local enrichment of TPE units.

The absence of such features at both micrometer (10×) and sub-micrometer (40×) scales indicates that the TPE moieties are molecularly dispersed/homogeneously distributed within the MMA matrix under both polymerization approaches, at least within the resolution limits of conventional optical microscopy.

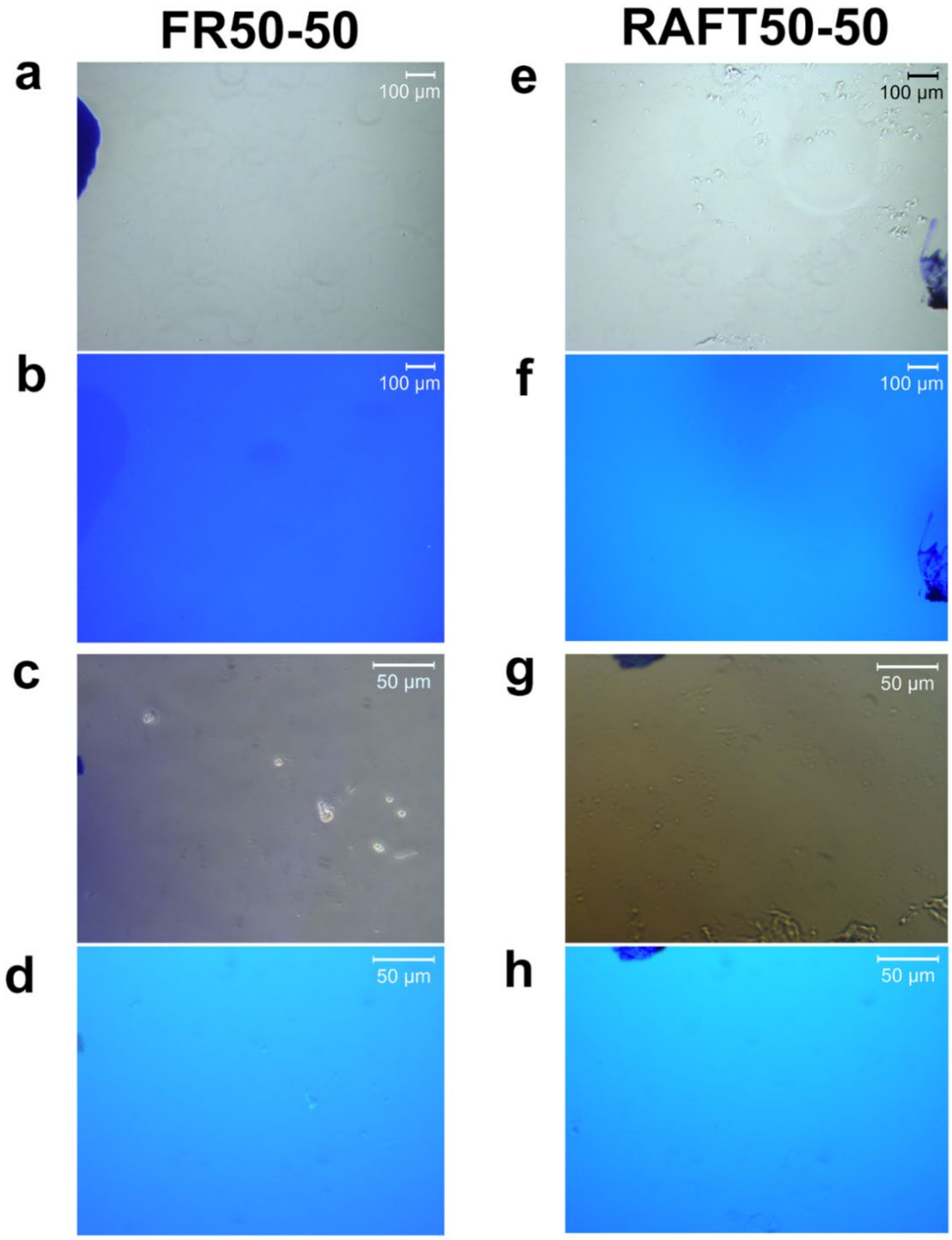

Supplementary Figure 26: Fluorescence and bright-field microscopy images of FR50-50 (left column) and RAFT50-50 (right column) copolymer films. Images were recorded at 10× (a,b,e,f) and 40× (c,d,g,h) magnification. Bright-field images (a,c,e,g) and fluorescence images under UV excitation (b,f,d,h) are shown for each condition. Scale bars: 100 µm (10×), 50 µm (40×).

3.3 Photostability Study of RAFT50-50 and FR50-50

To evaluate the impact of RAFT end-groups on the photostability of the LSC systems, we performed continuous irradiation experiments on RAFT50-50 and FR50-50 polymer films under a solar simulator (AM 1.5G, 100 mW/cm^2). Both systems were analyzed at regular intervals using UV–Vis and photoluminescence spectroscopy, and post-aging chemical changes were investigated with FTIR spectroscopy.

Under irradiation, both polymers exhibited a gradual loss of emission intensity as shown in **Supplementary Figure 27** (dashed lines). PL measurements revealed a 41% decrease in intensity for RAFT50-50 and 45% for FR50-50, indicating that the presence of RAFT end-groups does not significantly alter the rate of photobleaching. No shifts in emission peak positions or broadening were observed.

UV–Vis spectra (**Supplementary Figure 27 A,B**) showed a slight reduction in the absorption peak at 325 nm, attributed to the TPEMA chromophore. Additionally, a modest increase at ~250 nm was noted, which may be related to minor photoinduced changes in the polymer matrix or degradation side-products. Importantly, no new absorbance bands or visible yellowing of the films were observed (**Supplementary Figure 27 C,D**), and both remained optically clear.

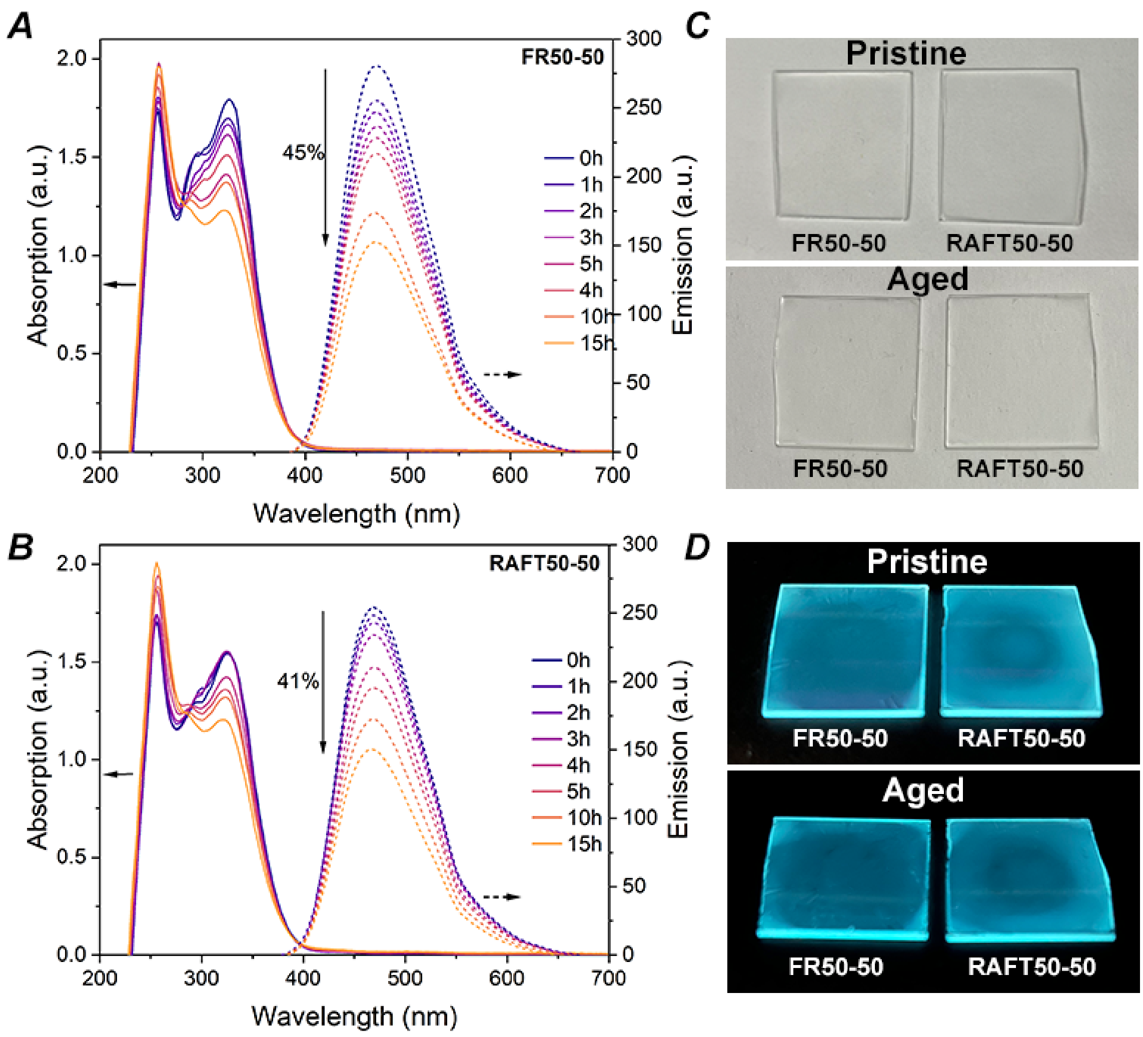

Supplementary Figure 27: Photostability assessment of FR50-50 and RAFT50-50 polymers under continuous solar simulator irradiation (AM 1.5G, 100 mW/cm²). UV–Vis absorption spectra (solid lines) and photoluminescence emission (dashed lines) of **A)** FR50-50 and **B)** RAFT50-50, measured at different irradiation times. **C)** Photographs of pristine and aged FR50-50 and RAFT50-50 polymer films under natural light. **D)** Corresponding images of the same films under UV light ($\lambda = 366$ nm),

To further investigate potential chemical degradation, we recorded FTIR spectra (**Supplementary Figure 28**) before and after irradiation. In both samples, the main ester carbonyl stretching band at ~1730 cm^{-1} shows only a minor decrease, indicating that the copolymer backbone remains largely stable under irradiation. For the RAFT50-50 sample, the broad band at ~3400 cm^{-1}, attributed to the –OH stretch of the terminal carboxylic acid (COOH) group present in the RAFT end-group, remains unchanged before and after aging. Subtle changes in the fingerprint region (1500-600 cm^{-1}) are observed for RAFT50-50, but without clear evidence of new bands related to sulfur- or aromatic-based degradation products (attributable to the RAFT agent).

In the FR50-50 sample, the ~3400 cm^{-1} band is initially less pronounced but increases after aging. This could be due to photo-oxidation leading to hydroxyl or carboxylic acid formation, or to increased moisture adsorption from minor surface polarity changes upon UV exposure.

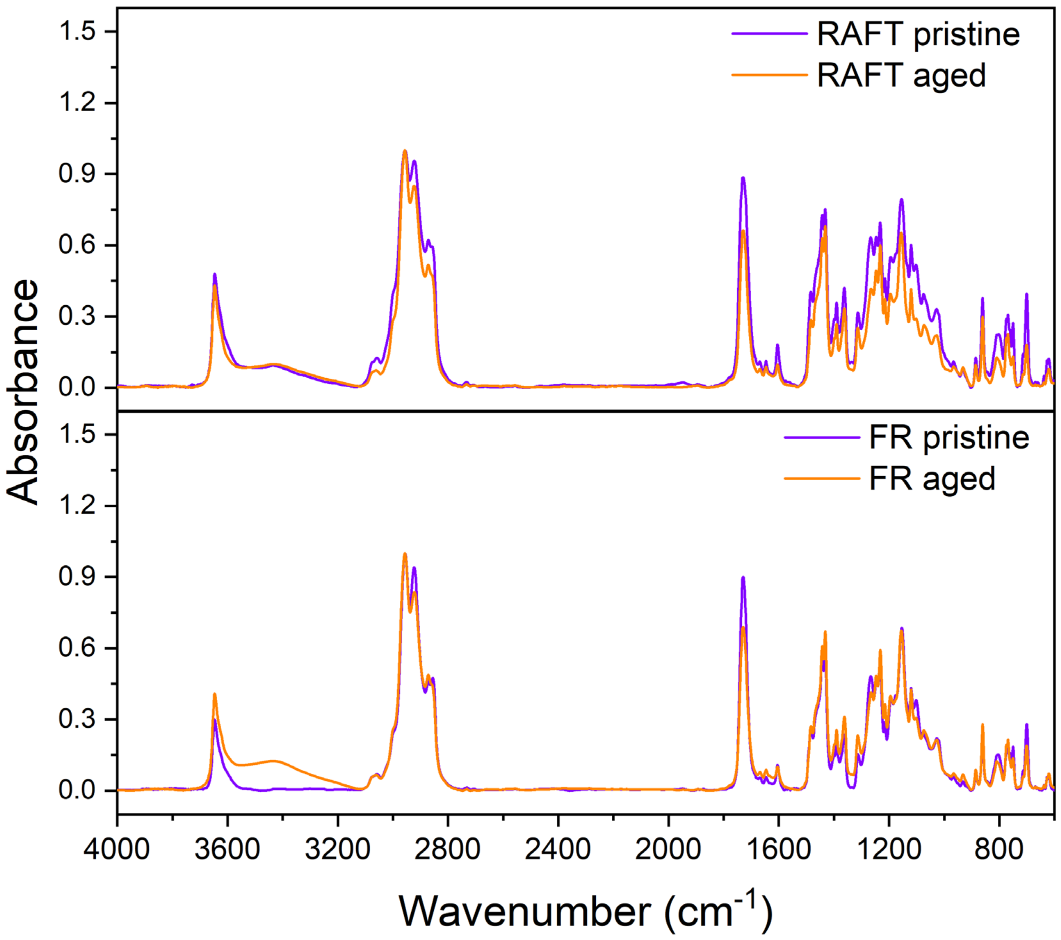

Supplementary Figure 28: FTIR spectra of pristine (purple) and aged (orange) polymer films after 15 h of continuous solar simulator irradiation (AM 1.5G).

Overall, FTIR results confirm that the presence of the RAFT end-groups does not introduce significant instability under prolonged light exposure when compared to free radical polymer, aligning with the photophysical measurements. In view of future application of these systems in real-life contexts, their long-term photostability could be significantly enhanced by employing commercially available photostabilizers (e.g., hindered-amine light stabilizers – HALS, or radical scavengers), which are typically included in acrylic formulations for outdoor use.

3.4 Emission vs Absorption

The fluorescence emission spectra spanned from 300 to 700 nm, with an emission peak red-shifting for increasing TPEMA molar content within the copolymer (**Figure 3 C, D**, main text). As previously noted, a distinct linear correlation is observed within the RAFT series when plotting the absorption maxima (λ_{abs}^{max}) against the emission maxima (λ_{em}^{max}). Conversely, this linear dependence is absent in the FR series (**Supplementary Figure 29**).

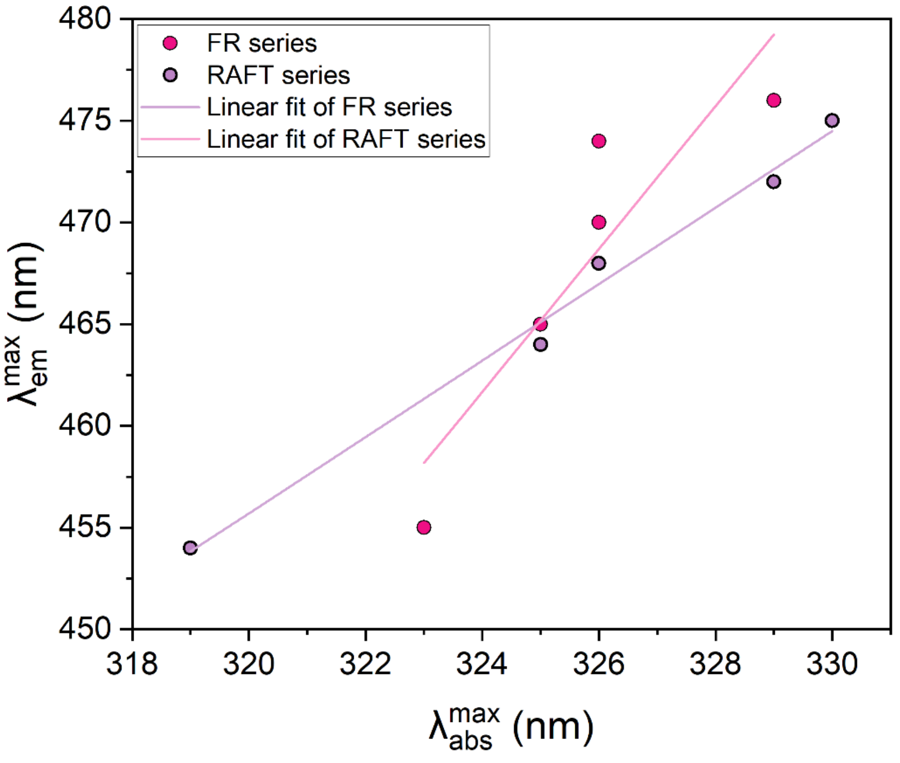

Supplementary Figure 29. Correlation between absorption maxima (λ_{abs}^{max}) and emission maxima (λ_{em}^{max}) for FR and RAFT series. Linear fit for FR series: y=3.51x-675.77; R^2=0.82. Linear fit for RAFT series: y=1.88x-145.80; R^2=0.99. 83.5 Photophysical properties of TPEMA monomer, RF and RAFT series

3.5 Photophysical properties of TPEMA monomer, RAFT and FR series

PLQY measurements of the films were performed by using a home-made integrating sphere according to the procedure reported elsewhere[7] with a SPEX 270M monochromator equipped with a LN2 cooled charge-coupled device, by exciting with a monochromated 450W Xe lamp at 370nm. The spectra are corrected for the instrument response. Time-resolved TCSPC measurements were obtained with PPD-850 single photon detector module by exciting with DeltaTime series DD-300 DeltaDiode and analyzed with the instrument Software DAS6. Average lifetimes are obtained by multi-exponential fittings as (**Supplementary Equation 12**):

$$\tau = \frac{\sum_i A_i t_i}{\sum_i A_i} \qquad (12)$$

where A_i and t_i are the 2- or 3-exponential fit parameters $\sum_i A_i t_i \; exp\left(\frac{t}{t_i}\right)$.

Supplementary Table 6. Photophysical properties of TPEMA monomer as cast film and diluted in THF solvent: fluorescence maximum position (λ_{em}^{max}); PLQY (λ_{ex}=370nm).

	λ_{em}^{max} (nm)	PLQY (%)
**TPEMA as cast film**	486	36
**TPEMA diluted in THF**	417	2.3

Supplementary Table 7. TPEMA monomer as cast film and diluted in THF solvent, FR series and RAFT series: average lifetimes (τ) obtained as $\tau = \sum_i \frac{A_i\, ti}{A_i}$ from 2-exponential and 3-exponential fits $\sum_i A_i\, e^{t/\tau}$ with parameters (t_1; t_2; t_3); (A_1; A_2; A_3); χ^2.

	**τ (ns)**	**t_1 (ns)**	**t_2 (ns)**	**t_3 (ns)**	**A_1 ($\cdot 10^{-2}$)**	**A_2 ($\cdot 10^{-2}$)**	**A_3 ($\cdot 10^{-2}$)**	**χ^2**
**TPEMA film**	1.98	0.376	2.504	5.307	5.524	5.392	1.794	1.162
**TPEMA in THF**	1.34	0.757	15.497	/	6.324	0.259	/	1.463
**FR1-99**	2.65	0.603	3.298	7.698	4.370	5.474	1.062	1.474
**FR10-90**	1.75	0.449	5.293	2.293	6.380	1.575	4.975	1.209
**FR20-80**	1.79	0.440	2.303	5.320	6.261	4.978	1.684	1.214
**FR50-50**	1.78	0.349	2.364	5.434	6.699	5.157	1.810	1.294
**FR75-25**	1.56	0.415	2.179	5.225	7.301	5.026	1.427	1.225
**RAFT1-99**	1.52	0.439	2.293	5.455	7.193	5.326	0.929	1.346
**RAFT10-90**	1.01	0.346	1.736	4.194	10.076	5.124	0.9465	1.199
**RAFT20-80**	1.56	0.364	2.174	5.172	7.549	5.042	1.625	1.258
**RAFT50-50**	1.49	0.352	2.129	5.142	7.979	4.989	1.603	1.339
**RAFT75-25**	1.59	0.375	2.217	5.276	7.365	4.977	1.576	1.204

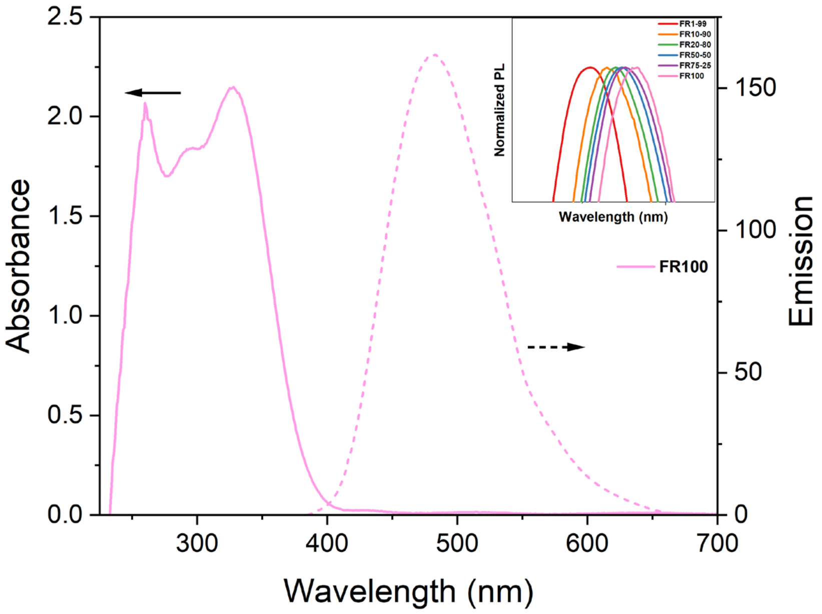

Supplementary Figure 30. UV-VIS and photoluminescence emission behavior of FR100, and inset: comparison between all PL normalized emissions of FR series.

4. Photonic and Photovoltaic Characterization

4.1. Radiative Overlap

To comprehensively understand the influence and extent of reabsorption losses on the performance of LSCs devices, the radiative overlap (RO) as a function of the luminophore concentration was evaluated. This parameter provides insight into the likelihood of reabsorption processes and quantifies the fraction of emitted light can be re-absorbed by the luminophore itself. It is calculated using **Supplementary Equation 13**:

$$RO = \frac{\int Em(\lambda)A(\lambda)d\lambda}{\int Em(\lambda)d\lambda} \cdot 100 \tag{13}$$

where Em(λ) is the emission spectrum and A(λ) is the absorption spectrum obtained through UV-vis absorption measurements. RO gives an estimation of the overlap between the absorption and the emission spectra of the luminescent material (**Supplementary Figure 12**); thus it should be as small as possible to ensure minimum reabsorption (RO→0 for an emitter with perfectly non-overlapping absorption and emission spectra).

As reported in **Supplementary Table 8**, a slight gradual increase in RO with increasing TPEMA molar concentration was observed in both series. Nevertheless, the RO values remained low, and the overall RO profile exhibited a quite flat trend (**Supplementary Figure 31**), denoting a high performance of such systems. This evidence is further supported by the large Stokes Shift values calculated and reported in this work, indicating a large separation between the absorption and emission bands which prevents the occurrence of most reabsorption events.

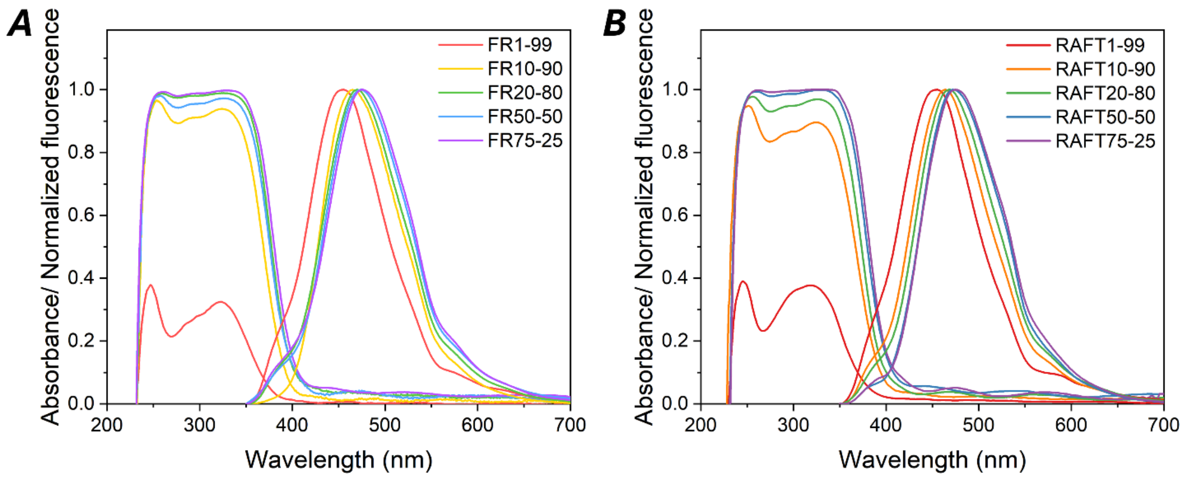

Supplementary Figure 31. Absorption (1-T(λ)) and normalized emission, for FR (A) and RAFT (B) series.

Supplementary Table 8. Radiative overlap (RO) calculated for both FR and RAFT systems.

**FR series**	**RO (%)**	**RAFT series**	**RO (%)**
**FR1-99**	0.55%	RAFT1-99	1.48%
**FR10-90**	1.40%	RAFT10-90	3.11%
**FR20-80**	4.24%	RAFT20-80	3.73%
**FR50-50**	3.95%	RAFT50-50	4.84%
**FR75-25**	5.06%	RAFT75-25	4.62%

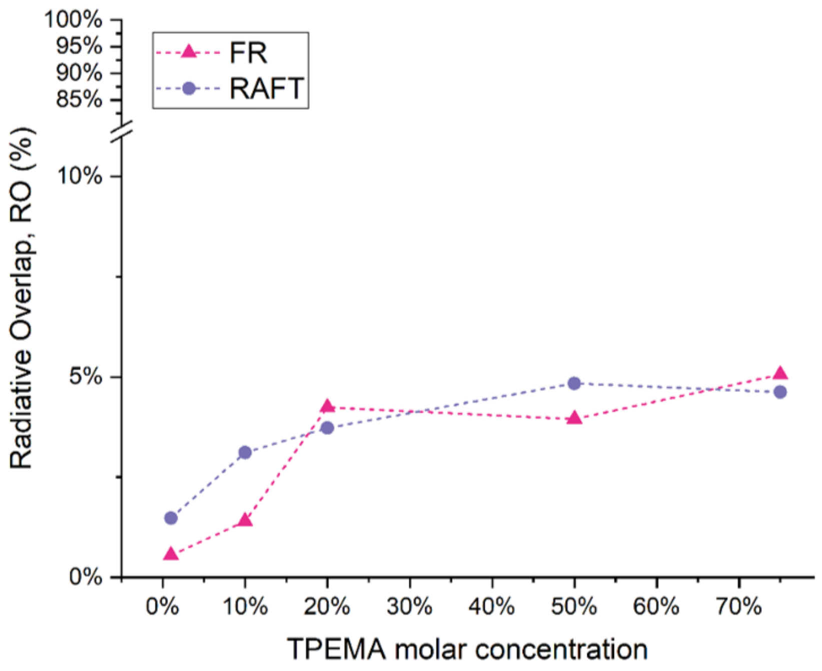

Supplementary Figure 32. Radiative overlap (RO) vs. TPEMA concentration in TPEMA-MMA systems.

4.2 Photonic Analysis

To characterize the optical performance of LSCs as photonic systems, two parameters were used, namely the external photon efficiency (η_{ext}) and the internal photon efficiency (η_{int}) (**Supplementary Equation 14** and **15**):

$$\eta_{ext} = \frac{N_{ph-out}}{N_{ph-in}} = \frac{N^{\underline{o}}\ of edge\ emitted\ photons}{N^{\underline{o}}\ of\ incident\ photons} = \frac{\sum_{i=1}^{4}\int_{300}^{800} P_{i(out)}(\lambda)\frac{\lambda}{hc}d\lambda}{\sum_{i=1}^{4}\int_{300}^{800} P_{(in)}(\lambda)\frac{\lambda}{hc}d\lambda} \qquad (14)$$

$$\eta_{int} = \frac{N_{ph-out}}{N_{ph-abs}} = \frac{N^{\underline{o}}\ of\ edge\ emitted\ photons}{N^{\underline{o}}\ of\ absorbed\ photons} = \frac{\sum_{i=1}^{4}\int_{300}^{800} P_{i(out)}(\lambda)\frac{\lambda}{hc}d\lambda}{\sum_{i=1}^{4}\int_{300}^{800} P_{(in)}(\lambda)\frac{\lambda}{hc}(1-10^{-A(\lambda)})d\lambda} \qquad (15)$$

where N_{ph-out} is the total number of edge-emitted photons summed over four edges (i = 1-4) of the LSC, N_{ph-abs} is the total number of photons absorbed by the LSC, and N_{ph-in} is the total number of photons incident on the top surface of the LSC. Also, h is Planck's constant (in J s) and c is the speed of light (in m s^{-1}). N_{ph-out} is obtained from the sum of the output power spectra, $P_{i(out)}(\lambda)$, measured for each edge of the LSC (in W nm^{-1}), where λ is the wavelength of light (in nm). $P_{in}(\lambda)$ is the input power spectrum from the solar simulator incident on the top surface of the LSC (in W nm^{-1}).

The absorption efficiency (η_{abs}), instead, is defined as the fraction of absorbed incident photons of the available photon flux. It is given by **Supplementary Equation 16**:

$$\eta_{abs} = \frac{\int_{0}^{\infty} S_{SO}\ (\lambda)\ [1-10^{-A(\lambda)}]\ d\lambda}{\int_{0}^{\infty} S_{SO}\ (\lambda) d\lambda} \qquad (16)$$

In **Supplementary Table 9**, all four-edges optical efficiencies for both series of TPEMA-MMA LSC systems are reported, measured in the 300-800 nm wavelength range under 1000 W·m^{-2} AM1.5G solar simulated light.

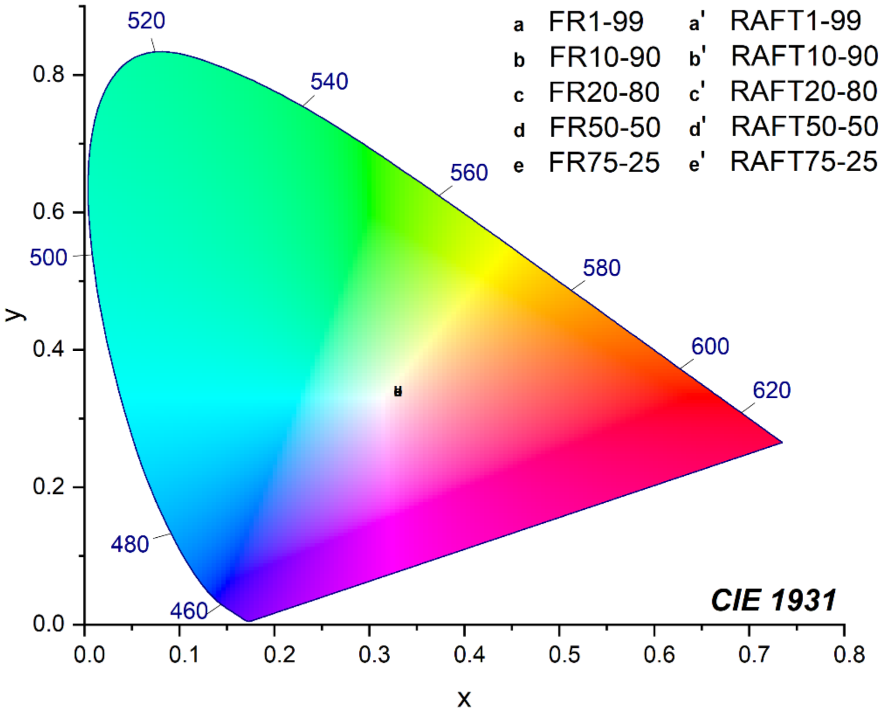

Supplementary Figure 33. CIE 1931 color space chromaticity diagram reporting the color coordinates of the thin-film LSCs based on TPEMA-MMA LSC systems.

Supplementary Table 9. Four-edges optical efficiencies of the fabricated TPEMA-MMA LSCs.

**Device**	η_{int} **(%)**	η_{ext} **(%)**	η_{abs} **(%)**
**FR1-99**	37.42 ± 1.87	1.13 ± 0.06	4.98 ± 0.25
**FR10-90**	23.56 ± 1.18	1.17 ± 0.06	5.66 ± 0.28
**FR20-80**	23.64 ± 1.18	1.23 ± 0.06	5.96 ± 0.30
**FR50-50**	31.05 ± 1.55	1.32 ± 0.07	4.93 ± 0.25
**FR75-25**	29.14 ± 1.46	1.35 ± 0.07	5.40 ± 0.27
**RAFT1-99**	34.89 ± 1.74	1.23 ± 0.06	5.87 ± 0.29
**RAFT10-90**	26.91 ± 1.35	1.31 ± 0.06	4.69 ± 0.23
**RAFT20-80**	31.19 ± 1.56	1.45 ± 0.07	5.36 ± 0.27
**RAFT50-50**	33.65 ± 1.68	1.61 ± 0.08	5.57 ± 0.28
**RAFT75-25**	33.22 ± 1.66	1.64 ± 0.08	5.79 ± 0.29

4.3 Power Conversion Efficiency, Average Visible-light Transmissivity (AVT) and Light Utilization Efficiency (LUE) of LSC-PV TPEMA-MMA systems

The power conversion efficiency (η_{dev}) was determined as the electrical power effectively extracted from the PV cells coupled with the LSC edges (P_{el}^{out}) relative to the optical input power hitting the top surface of the LSC (P_{opt}^{in}), as reported in **Supplementary Equation 17**:

$$\eta_{dev} = \frac{P_{el}^{out}}{P_{opt}^{in}} = \frac{V_{OC,LSC} \cdot I_{SC,LSC} \cdot FF}{P_{opt}^{in}\ A_{LSC}} \tag{17}$$

where FF, $I_{SC,LSC}$ and $V_{OC,LSC}$ are the fill factor, the short-circuit current and the open-circuit voltage of the LSC, respectively, P_{opt}^{in} is the incident solar power density (in mW cm^{-2}) and A_{LSC} is the front illuminated area of the LSC device (in cm^{2}). Measurements of the LSC-PV assemblies were conducted in series with a multimeter, under 1000 W·m^{-2} AM1.5G solar simulated light. Being the total active area of the PV cells equal to 4.8 cm^{2}, and the top surface of the LSC device equal to 25 cm^{2}, the final geometric gain considered was 5.21. The results retrieved from those tests are reported in **Supplementary Table 10**, including Open Circuit Voltage (V_{OC}), Short Circuit Current (I_{SC}), Fill Factor (FF), Power Conversion Efficiency of the LSC assembly (PCE_{LSC}), and Optical Efficiency (η_{opt}).

The average visible-light transmissivity (AVT), a figure of merit particularly useful in describing the transmissivity of the devices mediated with the photopic response of the human eye, was also evaluated for all copolymers made with FR and RAFT polymerization. The AVT was calculated with the following formula (**Supplementary Equation 18**):

$$AVT\% = \frac{\int T\%\,(\lambda)\ P(\lambda)\ S(\lambda) d\lambda}{\int\ P(\lambda)\ S(\lambda) d\lambda} \tag{18}$$

where T%(λ) is the transmissivity of the device, measured with a UV-Vis spectrophotometer, P(λ) is the photopic response of the human eye to light (i.e., the curve that describes which wavelengths are the cone cells most sensible to), and S(λ) is the AM 1.5G solar spectrum expressed as photon flux in $cm^{-2}s^{-1}$.

The product of AVT and η_{dev} gives the Light Utilization Efficiency (LUE):

$$\mathrm{LUE} = \mathrm{AVT} \cdot \eta_{dev} \tag{19}$$

which represents a more appropriate and holistic measure for comparing different devices in view of their applicability as transparent PV systems.

Values of AVT and LUE for all TPEMA-MMA based LSC systems investigated in this work are reported in **Supplementary Table 10**.

As already mentioned in the main text, the 50:50=TPEMA:MMA composition was selected as the best-performing one, and a total of four devices were created for each copolymer (FR50-50 and 50-50RAFT), adjusting the concentration from 5 wt% to 10 wt% and the deposition speed from 900 rpm to 600 rpm to maximize the thickness of the films.

The highest values obtained are evidenced in the table, demonstrating a device efficiency η_{dev} of 0.23% for the FR series (film thickness ~2.75 μm) and 0.29% for the RAFT series (film thickness ~2.29 μm), and LUE values equal to 0.21% (AVT~89%) and 0.25% (AVT~86%), respectively.

Supplementary Table 10. PV parameters and efficiencies of the fabricated TPEMA-MMA LSC devices: Open Circuit Voltage (V_{OC}), Short Circuit Current (I_{SC}), Fill Factor (FF), Power Conversion Efficiency of the LSC assembly (PCE_{LSC}), Optical Efficiency (η_{opt}), Average Visible Transmission (AVT) and Light Utilization Efficiency (LUE).

Sample	V_{OC} (V)	I_{SC} (mA)	FF (%)	PCE_{LSC} (%)	η_{opt} (%)	AVT (%)	LUE (%)
**a**	2.159	3.904	0.583	0.197	1.300	90.10	0.177
**b**	2.159	3.808	0.612	0.201	1.302	89.65	0.180
**c**	**2.179**	**4.533**	**0.589**	**0.233**	**1.562**	**89.35**	**0.208**
**d**	2.140	4.564	0.581	0.227	1.485	89.21	0.203
**a'**	2.142	3.802	0.602	0.196	1.326	90.31	0.177
**b'**	2.109	4.121	0.594	0.206	1.370	90.50	0.186
**c'**	2.079	4.337	0.581	0.210	1.408	89.89	0.189
**d'**	**2.173**	**5.316**	**0.619**	**0.286**	**1.927**	**85.95**	**0.246**

Sample: 5 wt% of copolymer in $CHCl_3$, at 900 rpm → a=FRP, a'=RAFT; 5 wt% of copolymer in $CHCl_3$, at 600 rpm → b=FRP, b'=RAFT; 10 wt% of copolymer in $CHCl_3$, at 900 rpm → c=FRP, c'=RAFT; 10 wt% of copolymer in $CHCl_3$, at 600 rpm → d = FRP, d' = RAFT.

To facilitate meaningful benchmarking, we have compiled the available reported figures of merit for recent colorless or semi-transparent LSC systems in **Supplementary Table 11**. Where possible, these values follow the standardized reporting protocols proposed Yang et al. and Debije et al.[8,9] We specifically focused on systems employing luminophores that absorb outside the visible range (i.e., in the UV or NIR), thereby producing colorless devices. Where complete performance metrics were not reported in the original papers, the relevant fields are noted as "n/a."; no estimations were made due to the lack of raw data. We note that some earlier valuable

studies on colorless LSCs, for example using different types of QDs[10], organic dyes[11], or organometallic complexes[12] predate these protocols and do not provide sufficient information to extract all relevant performance metrics reliably.[8,9] Consequently, these have not been included in the summary table. Nonetheless, the selected examples illustrate the state of the art in genuinely colorless LSCs and support the competitive performance of the present work.

Supplementary Table 11: Comparison of key performance parameters (η_{ext}, η_{int}, η_{dev}, AVT, LUE) for recent colorless luminescent solar concentrators (LSCs).

	Colorless LSCs	**AVT %**	**LUE%**	**η_{ext} %**	**η_{int} %**	**η_{dev} %**	**year**	**Ref.**
**1**	This work: polymeric AIEgens	86	0.25	1.64	33	0.29 [a]	2025	
**2**	ZnO QDs	80.3	n/a	n/a	n/a	up to 3.80 [a] and 5.45 [b]	2024	13
**3**	NIR-bacteriochlorophyll	73-94	n/a	1	n/a	0.04	2025	14
**4**	AIEgens	n/a	n/a	n/a	n/a	0.32[a]	2015	15
**5**	SiQDs	86	*	*	n/a	0.27 [a]	2022	16
**6**	Ultraviolet and Near-Infrared Dual-Band	75	2.6	n/a	n/a	3	2021	17

[a] calculated with mc-Si solar cells, [b] calculated with OPV solar cells, * efficiencies calculated based on other type of calculation

Supplementary References

1. Nguyen, G., Nicole, D., Swistek, M., Matlengiewicz, M. & Wiegert, B. Sequence distribution of the methyl methacrylate-ethyl acrylate copolymer by 13 C n.m.r. spectroscopy. *Polymer* **38**, 3455–3461 (1997).

2. A. S. Brar, M. Mukherjee, S. K. Chatterjee, Compositional Sequence Determination of Acrylamide/Alkyl Acrylate Copolymers by NMR Spectroscopy, *Polymer Journal* **30**, 664-670 (1998)

3. Fuentes Estevez, G., Valdes Lizama, O., Zaldivar Silva, D., Aguero, L. & Katime, I. A New Statistical Point Of View To Choose A Better Linear Model For Reactivity And Microstructure Analysis In HEMA/furfuryl Acrylate Copolymerization Process. *Adv. Mater. Lett.* **4**, 534–542 (2013).

4. Boulding, N. A., Millican, J. M. & Hutchings, L. R. Understanding copolymerisation kinetics for the design of functional copolymers *via* free radical polymerisation. *Polym. Chem.* **10**, 5665–5675 (2019).

5. Abdollahi, H., Najafi, V. & Amiri, F. Determination of monomer reactivity ratios and thermal properties of poly(GMA-co-MMA) copolymers. *Polym. Bull.* **78**, 493–511 (2021).

6. Fox, T. G. & Flory, P. J. The glass temperature and related properties of polystyrene. Influence of molecular weight. *J. Polym. Sci.* **14**, 315–319 (1954).

7. Moreau, J. *et al.* Highly Emissive Nanostructured Thin Films of Organic Host–Guests for Energy Conversion. *ChemPhysChem* **10**, 647–653 (2009).

8. Yang, C. *et al.* Consensus statement: Standardized reporting of power-producing luminescent solar concentrator performance. *Joule* **6**, 8–15 (2022).

9. Debije, M. G., Evans, R. C. & Griffini, G. Laboratory protocols for measuring and reporting the performance of luminescent solar concentrators. *Energy Environ. Sci.* **14**, 293–301 (2021).

10. Meinardi, F. *et al.* Highly efficient large-area colourless luminescent solar concentrators using heavy-metal-free colloidal quantum dots. *Nature Nanotech* **10**, 878–885 (2015).

11. Bellina, F. *et al.* Colourless p -phenylene-spaced bis-azoles for luminescent concentrators. *Dyes and Pigments* **134**, 118–128 (2016).

12. Fiorini, V. *et al.* Colourless luminescent solar concentrators based on Iridium(III)-Phosphors. *Dyes and Pigments* **193**, 109532 (2021).

13. Fimbres-Romero, M. D. J., Flores-Pacheco, Á., Álvarez-Ramos, M. E. & Lopez-Delgado, R. Transparent and Colorless Luminescent Solar Concentrators Based on ZnO Quantum Dots for Building-Integrated Photovoltaics. *ACS Omega* **9**, 28008–28017 (2024).

14. Correia, S. F. H. *et al.* Transparent nature-based luminescent solar concentrator with NIR emission and integrated thermal sensing. *J. Mater. Chem. A* **13**, 11886–11898 (2025).

15. Banal, J. L. *et al.* A Transparent Planar Concentrator Using Aggregates of *gem* -Pyrene Ethenes. *Advanced Energy Materials* **5**, 1500818 (2015).

16. Han, S. *et al.* Luminescence-guided and visibly transparent solar concentrators based on silicon quantum dots. *Opt. Express* **30**, 26896 (2022).

17. Yang, C. *et al.* Ultraviolet and Near-Infrared Dual-Band Selective-Harvesting Transparent Luminescent Solar Concentrators. *Advanced Energy Materials* **11**, 2003581 (2021).
