# Supplementary material for: Tailoring aggregation-induced emission in luminescent solar concentrators through controlled polymerization
Source: Commun Chem. 2025 Oct 17;8:312. doi: 10.1038/s42004-025-01700-1 (PMC12534600; doi:10.1038/s42004-025-01700-1)
Supplement: Supplementary file 2 — Photovoltaic reporting [file 42004_2025_1700_MOESM2_ESM.pdf]

nature portfolio

Corresponding author(s): Gianmarco Griffini

Solar Cells Reporting Summary

Nature Portfolio wishes to improve the reproducibility of the work that we publish. This form is intended for publication with all accepted papers reporting the characterization of photovoltaic devices and provides structure for consistency and transparency in reporting. Some list items might not apply to an individual manuscript, but all fields must be completed for clarity.

For further information on Nature Research policies, including our data availability policy, see Authors & Referees.

Experimental design

Please check the following details are reported in the manuscript, and provide a brief description or explanation where applicable.

1. Dimensions

Area of the tested solar cells

☒ Yes — 4 commercially available 2.2 x 0.6 cm^2 mc-Si solar cells connected in series

☐ No — *Explain why this information is not reported/not relevant.*

Method used to determine the device area

☒ Yes — data sheet of the provider (IXOLAR SolarBIT KXOB25-12X1L, IXYS)

☐ No — *Explain why this information is not reported/not relevant.*

2. Current-voltage characterization

Current density-voltage (J-V) plots in both forward and backward direction

☐ Yes

☒ No — Not applicable. J–V curves were recorded in the forward scan direction (–0.5 to 2.5 V). Reverse scans were not included, as forward scans represent the standard characterization protocol for this type of experiment. The solar cells used are commercially available devices.

Voltage scan conditions

☒ Yes — Scan direction: Forward (- 0.5 → +2.5 V)
Voltage step: 40 mV
dwell time: 700 ms.

☐ No — *Explain why this information is not reported/not relevant.*

Test environment

☒ Yes — J-V measurements were performed under a calibrated solar simulator (AM1.5G, 1000 W m-2).

☐ No — *Explain why this information is not reported/not relevant.*

Protocol for preconditioning of the device before its characterization

☐ Yes — *Provide a description of the protocol.*

☒ No — No special preconditioning was applied; devices were measured in the as-fabricated state.

Stability of the J-V characteristic

☐ Yes — *Provide a description of the method used. The stability of the J-V characteristic can be verified with time evolution of the maximum power point or with the photocurrent at maximum power point; see ref. 5 for details.*

☒ No — Current–voltage (J–V) characteristics were obtained from repeated measurements (each device measured at least three times) to ensure reproducibility. Commercial multicrystalline silicon (mc-Si) solar cells were used; stability testing of J–V characteristics was not performed as it is beyond the scope of this study.

3. Hysteresis or any other unusual behaviour

Description of the unusual behaviour observed during the characterization

☐ Yes — *Provide a description of hysteresis or any other unusual behaviour observed during the characterization.*

☒ No — beyond the scope of this study

Related experimental data

☐ Yes — *Provide a description of the related experimental data.*

☒ No — n/a

4. Efficiency

External quantum efficiency (EQE) or incident photons to current efficiency (IPCE)

☐ Yes — *Provide a description of the technique used.*

☒ No — Not applicable. Our study is focused on photonic characterization of luminescent solar concentrators.

A comparison between the integrated response under the standard reference spectrum and the response measure under the simulator

☐ Yes
☒ No

State where this information can be found in the text.

Not applicable - see previous comment.

For tandem solar cells, the bias illumination and bias voltage used for each subcell

☐ Yes
☒ No

Provide a description of the measurement conditions.

n/a

5. Calibration

Light source and reference cell or sensor used for the characterization

☒ Yes
☐ No

Calibration was verified using a JRC-calibrated certified reference silicon solar cell (PVM689, PV Measurements Inc.) and a calibrated powermeter (Nova II, MKS Ophir) to ensure correct light intensity.

Explain why this information is not reported/not relevant.

Confirmation that the reference cell was calibrated and certified

☒ Yes
☐ No

Confirmed.

Explain why this information is not reported/not relevant.

Calculation of spectral mismatch between the reference cell and the devices under test

☐ Yes
☒ No

Provide a value of the spectral mismatch and/or a description of how it has been taken into account in the measurements.

Not applicable.

6. Mask/aperture

Size of the mask/aperture used during testing

☒ Yes
☐ No

A 4.9 x 4.9 cm2 mask was applied over the LSC area to prevent direct irradiation of the photovoltaic cells.

Explain why this information is not reported/not relevant.

Variation of the measured short-circuit current density with the mask/aperture area

☒ Yes
☐ No

Variations in short-circuit current density were observed when altering the mask/aperture area, as expected, since the mask reduces the contribution of photons that would otherwise directly strike the solar cells. Data reported are obtained from masked devices.

Explain why this information is not reported/not relevant.

7. Performance certification

Identity of the independent certification laboratory that confirmed the photovoltaic performance

☐ Yes
☒ No

Identify the independent certification laboratory.

Certification is not applicable. Device performance was not independently certified, as the scope of this work is proof-of-concept demonstration.

A copy of any certificate(s)

☐ Yes
☒ No

Certificate copies should be provided in the Supplementary information. Please state the supplementary item number.

n/a

8. Statistics

Number of solar cells tested

☒ Yes
☐ No

In total, 32 solar cells were tested (4 cells per device). Measurements were highly consistent across devices.

Explain why this information is not reported/not relevant.

Statistical analysis of the device performance

☐ Yes
☒ No

State where this information can be found in the text.

No formal statistical analysis was performed, as statistical treatment is beyond the scope of this materials-focused study.

9. Long-term stability analysis

Type of analysis, bias conditions and environmental conditions

☐ Yes
☒ No

Provide a description of the type of analysis, bias conditions and environmental conditions (e.g. illumination type, temperature, atmosphere humidity, encapsulation method, preconditioning temperature, bias) for each long-term stability analysis carried out; see ref. 7 and 8 for details.

Long-term stability testing of the LSC/PV was not performed. Though the LSCs were studied and tested from a photophysical point of view. The work is limited to initial LSC device performance evaluation; stability under continuous operation will be addressed in future studies.
